# Four new complete mitochondrial genomes of Gobioninae fishes (Teleostei: Cyprinidae) and their phylogenetic implications

**DOI:** 10.7717/peerj.16632

**Published:** 2024-01-19

**Authors:** Yongtao Tang, Wenwen Ma, Xin Chen, Guoxing Nie, Chuanjiang Zhou

**Affiliations:** 1 College of Fisheries, Engineering Technology Research Center of Henan Province for Aquatic Animal Cultivation, Henan Normal University, Xinxiang, Henan province, The People’s Republic of China; 2 College of Life Sciences, Henan Normal University, Xinxiang, Henan Province, China

**Keywords:** Gobioninae, Mitochondrial genome, Phylogenetic relationships

## Abstract

The subfamily Gobioninae is one of the most diverse fish groups within Cyprinidae. Their taxonomy and phylogenetic relationships are not completely resolved. In this study, the complete mitochondrial genomes (mitogenome) of four Gobioninae species (*Microphysogobio elongatus*, *Microphysogobio chinssuensis*, *Gobio rivuloides* and *Rhinogobio nasutus*) were sequenced and compared. The mitogenomes of four species ranges from 16603 bp to 16609 bp in length, consisting of 13 protein-coding genes (PCGs), 22 tRNA genes, two rRNA genes, and a control region. Most PCGs had significant codon usage bias. Except for the tRNA^Ser^ (GCT), all the nucleotide substitutions of tRNA loops higher than the stems could fold into a stable secondary structure. The nucleotide compositions of Gobioninae mitogenome were biased toward A/T, and NAD4 was subjected to low purification selection and had a faster evolution rate among 13 PCGs. Bayesian inference and maximum likelihood phylogenetic analyses showed the consistent results. The four sequenced species clustered together with their congener species. However, more samples and mitogenome data are needed to untangle the phylogenetic relationships among genera *Microphysogobio*, *Romanogobio*, *Hugobio*, *Biwia* and *Platysmacheilus.*

## Introduction

Gobioninae (also called gudgeons) is a subfamily of small and medium-sized Cyprinid fishes distributed across Asia and Europe. There are 29 genera and more than 200 Gobioninae species (Rainboth, 1991; Baˇnaˇrescu, 1992; Yang et al., 2006; Liu, Yang & Tang, 2010; Tang et al., 2011; Van Der Laan, Eschmeyer & Fricke, 2014; Nelson, Grande & Wilson, 2016). Gobioninae fishes present great species diversity due to their different life history traits, ecologies and living habitats (Baˇnaˇrescu, 1992; Yue, 1998; Li, 2017). For example, most species live in freshwater environments, while some species of *Gobio*, *Romanogobio, Microphysogobio*, and *Squalidus* (*e.g., G. gobio, S. minor*) occur in both freshwater and brackish water habitats (Van Der Laan, Eschmeyer & Fricke, 2014). Most Gobioninae species are benthic and occur in areas with sand/cobble bottoms, but some genera are medial dwellers or live in low-water environments (Li, 2017; Baˇnaˇrescu & Nalbant, 1973; Baˇnaˇrescu & Coad, 1991).

Early studies of the species relationships within Gobioninae were based on morphology and did not produce consistent results (Rainboth, 1991; Baˇnaˇrescu, 1992; Naseka, 1996; Yu & Yue, 1996; Yu & Liu, 2011). Recent studies using molecular data provided some new insights into the phylogenetic relationships among Gobioninae species. Four monophyletic groups were found based on the mitochondrial Cyt *b* gene (Yang et al., 2006; Liu, Yang & Tang, 2010; Kim et al., 2013). However, only three lineages were detected when four genes (COI, Cyt *b*, RAG1, RH) were used (Tang et al., 2011). Zhao et al. (2016) reconstructed the phylogenetic relationship for 22 Gobioninae species using mitochondrial genome (mitogenome) data, which significantly improved the node support of the tree. However, due to the lack of a mitogenome of representative samples from most genera, the relationship among them is still not fully examined. Many phylogenetic studies of Gobioninae note that increasing the number of species and molecular markers will improve our understanding of their phylogenetic relationships (Yang et al., 2006; Liu, Yang & Tang, 2010; Tang et al., 2011; Zhao et al., 2016; Kim et al., 2013).

Because of their rapid evolutionary rate and disparity in evolutionary rate, mitochondrial genes are usually considered suitable for phylogenetic research at various taxonomic levels (Brown, George & Wilson, 1979). In studies of fish phylogeny, using the whole mitogenome as one molecular marker is a common and effective approach because of their conservative gene content, maternal inheritance, large copy numbers, small gene gaps, and absence of introns. Moreover, the number of published Gobioninae mitogenomes has increased, and these provide valuable data for studies on species identification, population genetics and evolutionary relationships (Kim et al., 2013; Zhao et al., 2016).

In previous studies, only partial mitogenome genes or limited samples were used to construct phylogenetic trees (Yang et al., 2006; Liu, Yang & Tang, 2010; Tang et al., 2011; Zhao et al., 2016; Kim et al., 2013). Therefore, a comprehensive phylogenetic investigation of Gobioninae based on denser taxon and mitochondrial sequence coverage is needed to construct a robust phylogenetic relationship of this group. Here, a great deal of complete mitogenomes containing 85 Gobioninae species and 31 representative species of other subfamilies in Cypriniformes were downloaded from the National Center for Biotechnology Information (NCBI, https://www.ncbi.nlm.nih.gov/) (Table S1). Moreover, we newly sequenced four mitogenomes of Gobioninae species, *Microphysogobio elongatus*, *Microphysogobio chinssuensis*, *Gobio rivuloides* and *Rhinogobio nasutus*, to further extend the molecular data for reconstructing the phylogeny of Gobioninae. Three of these four species, *M. elongatus*, *M. chinssuensis*, and *G. rivuloides,* are widespread species, and *R. nasutu* is a local species that only occurs in the Yellow River (Li, 2017). Furthermore, we compared the characteristics of the mitogenome, including genome organization, tRNA secondary structures and codon usage patterns.

## Materials and methods

### Sampling, identification, DNA extraction, PCR amplification and sequencing

Specimens were collected from the Yellow River and Haihe River, Henan Province, China (Table 1) and were identified based on morphological characters as described by Luo, Yue & Chen (1977) and Yue (1998). Total genomic DNA was extracted from the muscle tissue using a standard phenol–chloroform method (Sambrook & Russell, 2001). The final concentration and purity were measured on a Nanodrop 2000 system.

**Table 1:** Details of sample collection information about this study (sample collection sites including river system, sample date, and collector).

Species	Collecting sites	Collecting times	Collector
*Microphysogobio elongatus*	The Yellow River, Mengzhou, Henan Province, China	8, May, 2017	The authors
*M. chinssuensis*	Hai River, Anyang, Henan Province, China	20, Jan, 2019	The authors
*Gobio rivuloides*	The Yellow River, Lingbao County, Sanmenxia, Henan Province, China	14, Aug, 2016	The authors
*Rhinogobio nasutus*	The Yellow River, Zhongmou County, Zhenzhou, Henan Province, China	9, Aug, 2019	The authors

To obtain more reliable and precise contigs to assemble the complete mitogenome, Sanger sequencing based on multiprimer PCR amplification was used. PCR amplification was performed on the qualified samples, and the primer design was based on the published nucleotide sequences of multiple genera, including *Gobio, Microphysogobio,* and *Rhinogobio.* Twenty-two pairs of universal primers and three pairs of species-specific primers were designed using Primer Premier 5.0 software (Table S2) (Lalitha, 2000). To ensure the accuracy of mitogenome assembly, we used two adjacent regions with 100 bp overlap. PCRs were conducted under the following conditions: predenaturation at 95 °C for 5 min; 35 cycles of 95 °C for 30 s, annealing at 50–55 °C for 30 s, and extension at 72 °C for 90 s; and a final extension at 72 °C for 8 min. The PCR products were determined by 1.5% agarose gel electrophoresis and were submitted to Sangon Biotech (Shanghai) Co., Ltd. for sequencing.

### Mitogenome assembly, annotation, and sequence analyses

Seqman software was used to check the peak plots of sequences, and poor-quality parts were trimmed on both sides (Swindel & Plasterer, 1997). The selection of some individual sites was manually corrected, and the sequences were assembled to obtain a series of short sequences. The output short sequences were spliced using the Seqman program Lasergene 7.0 to finish the preliminary assembly of the complete mitogenome (Burland, 2000). Through a comparison with the mitogenomes of Gobioninae fishes published on NCBI, the positions of the initial tRNA (tRNA^Ile^) and the control region were determined, and the complete and ordered mitogenomes were obtained.

The assembled mitogenome was annotated using the vertebrate genetic code in the MITOS webserver (http://mitos.bioinf.uni-leipzig.de/index.py) (Bern et al., 2013). For greater accuracy, the 22 tRNA genes were reidentified, and their secondary structures were predicted using tRNAscan-SE 1.21 (Lowe & Chan, 2016). For further determination of the accurate gene boundaries, the 13 protein-coding genes (PCGs) were translated into amino acids using the Editseq program Lasergene 7.0 on the basis of the vertebrate mitochondrial genetic code (Burland, 2000). The ribosomal RNAs (rRNAs) were identified by alignment with the homologous genes of other Gobioninae species. To verify the reliability of the annotated results, CSBs were identified by comparing the recognition sites with other Cyprinid species, and tandem repeat elements were detected by the Tandem Repeats Finder program (http://tandem.bu.edu/trf/trf.html) (Benson, 1998) in the control region.

The circular maps of the four new Gobioninae mitogenomes were drawn by CGView Server (Grant & Stothard, 2008). Nucleotide composition, codon usage, amino acid composition and relative synonymous codon usage (RSCU) values were calculated by MEGA 7.0 software (Sudhir, Glen & Koichiro, 2016). Nucleotide bias was calculated according to the following formulas: AT skew = [A − T]/[A + T] and GC skew = [G − C]/[G + C] (Perna & Kocher, 1995). Nucleotide diversity, the ratio of nonsynonymous substitution (Ka), synonymous substitution (Ks), and the ratio of Ka/Ks for all PCGs were calculated by DnaSP 5.1 (Librado & Rozas, 2009). In addition, effective codon usage statistics (ENC) and the codon adaptation index (CAI) were measured using CodonW 1.4.2 (Peden, 2000).

### Phylogenetic analyses

To study the phylogenetic relationships of Gobioninae, we downloaded 116 mitogenome sequences from the GenBank database, including 85 Gobioninae species from 27 genera and 31 sequences of other Cypriniformes species. *Myxocyprinus asiaticus* and *Cobitis lutheri* were used as outgroups (Table S1) according to previous research (Zhao et al., 2016). Among the mitochondrial genes, 13 PCGs and two rRNAs were used for phylogenetic analyses. Sequence alignments were performed within MEGA 7.0 (Sudhir, Glen & Koichiro, 2016) for the 13 PCGs and within MAFFT under the Q-INS-i algorithm (Katoh & Standley, 2016) for the two rRNAs. Then, individual genes were concatenated by Phylosuite (Zhang et al., 2020; Huelsenbeck & Ronquist, 2001). The best partitioning schemes and corresponding nucleotide substitution models for each dataset were selected by PartitionFinder v.1.1.1 (Lanfear et al., 2012) with the Bayesian Information Criterion (BIC) algorithm under a greedy search (Table S3). To obtain more reliable data and the different features of PCGs and the two rRNAs, phylogenetic analyses of two datasets were constructed by the maximum likelihood (ML) and Bayesian inference (BI) method in this study: (1) sequences of 13 PCGs; (2) sequences of 13 PCGs and 2 rRNAs. ML analysis and BI analysis were implemented in RAxML-HPC2 and MrBayes 3.2.6, respectively, running on the CIPRES Science gateway (Stamatakis, 2006; Huelsenbeck & Ronquist, 2001; Miller, Pfeiffer & Schwartz, 2010). Under ML analysis, bootstrap support (BS) was assessed using 1,000 ultrafast bootstrap replicates. BI analysis was executed with 10 million generations with four chains, sampling trees every 1,000 generations. After discarding the first 25% of the yielded trees as a burn-in phase, the 50% majority-rule consensus trees were estimated for the remaining trees. Branch support was assessed using posterior probabilities (PP).

## Results and discussion

### Features of the newly sequenced mitochondrial genomes

**Figure 1:**
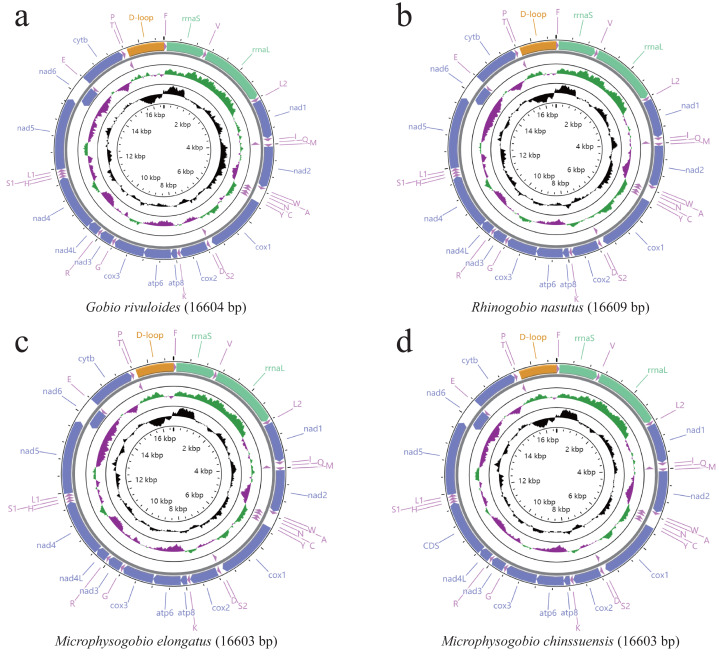
(A–D) Gene content and composition maps of four Gobioninae mitochondrial genomes inferred from the MITOS webserver (http://mitos.bioinf.uni-leipzig.de/index.py).

The mitogenomes of the four Gobioninae species were typical circular molecules with lengths of 16,603 bp for *M. elongate* and *M. chinssuensis*, 16,604 bp for *G. rivuloides* and 16,609 bp for *R. nasutus* (Table 2). The lengths, gene content and gene arrangement of the four Gobioninae mitogenomes were highly typical for teleost fishes. The typical 37 genes were detected in the new mitogenomes, including 13 typical PCGs, 22 transfer RNA genes (tRNAs), two rRNAs and two noncoding regions (Satoh et al., 2016; Hwang, Byeon & Lee, 2014). Among them, 28 genes were encoded on the heavy strand (H strand), and the remaining genes were located on the light strand (L strand) (Fig. 1). All the sequences of this study had gene arrangements that were identical to other published mitogenomes of Cyprinid fishes (Hwang, Byeon & Lee, 2014). The nucleotide composition of four newly sequenced Gobioninae fishes revealed a strong A + T bias in the whole mitogenome ranging from 55.4% in *G. rivuloides* to 57.8% in *R. nasutus*. The AT skew of the newly sequenced mitogenomes varied from 0.04 (*G. rivuloides*) to 0.08 (*R. nasutus*), and the GC skew varied from −0.24 (*R. nasutus*) to −0.18 (*G. rivuloides*) (Table 3). Similar to other Gobioninae fishes, the complete mitogenome sequences were biased toward A + T and showed positive A skew and C skew. The phenomenon of specific base composition bias has been inferred as the result of asymmetrical directional mutation pressure (Reyes et al., 1998; Yang et al., 2018).

**Table 2:** Detailed information about gene content and composition of the four newly determined Gobioninae mitochondrial genomes. The statistical results of the newly determined mitogenomes of *G. rivuloides*, *R. nasutus*, *M. chinssuensis*, and *M. elongate*.

Genes	Position		Size (bp)	Intergenic length	Amino acids	Start codon	Stop codon	Anticodon	Strand
	Fom	To							
tRNA^Phe^	1/1/1/1	69/69/69/69	69/69/69/69					GAA/GAA/GAA/GAA	H
12SrRNA	70/70/70/70	1028/1029/1027/1028	959/960/958/959						H
tRNA^V al^	1029/1030/1028/1029	1100/1101/1099/1100	72/72/72/72					TAC/TAC/TAC/TAC	H
16SrRNA	1101/1102/1100/1101	2793/2794/2790/2790	1693/1693/1691/1690						H
tRNA^Leu(UUR)^	2794/2795/2791/2791	2869/2870/2866/2866	76/76/76/76					TAA/TAA/TAA/TAA	H
NAD1	2871/2872/2868/2868	3845/3846/3842/3842	975/975/975/975	1/1/1/1	324/324/324/325	ATG/ATG/ATG/ATG	TAG/TAA/TAG/TAG		H
tRNA^Ile^	3850/3851/3847/3847	3921/3922/3918/3918	72/72/72/72	4/4/4/4				GAT/GAT/GAT/GAT	H
tRNA^Gln^	3920/3921/3917/3917	3990/3991/3987/3987	71/71/71/71	-1/-2/-2/-2				TTG/TTG/TTG/TTG	L
tRNA^Met^	3992/3993/3989/3989	4060/4061/4057/4057	69/69/69/69	1/1/1/1				CAT/CAT/CAT/CAT	H
NAD2	4061/4062/4058/4058	5105/5107/5103/5103	1045/1046/1046/1046		348/348/348/348	ATG/ATG/ATG/ATG	T–/TA-/TA-/TA-		H
tRNA^Trp^	5106/5108/5104/5104	5176/5179/5174/5174	71/72/71/71					TCA/TCA/TCA/TCA	H
tRNA^Ala^	5179/5182/5177/5177	5247/5250/5245/5245	69/69/69/69	2/2/2/2				TGC/TGC/TGC/TGC	L
tRNA^Asn^	5249/5252/5247/5247	5321/5324/5319/5319	73/73/73/73	1/1/1/1				GTT/GTT/GTT/GTT	L
OL	5323/5327/5322/5322	5353/5356/5351/5351	31/30/30/30	1/2/2/2					H
tRNA^Cys^	5352/5355/5351/5351	5419/5422/5418/5418	68/68/68/68	-1/-2/-1/-1				GCA/GCA/GCA/GCA	L
tRNA^Tyr^	5422/5425/5421/5421	5492/5495/5490/5490	71/71/70/70	2/2/2/2				GTA/GTA/GTA/GTA	L
CoX1	5494/5497/5492/5492	7044/7047/7042/7042	1551/1551/1551/1551	1/1/1/1	516/516/516/517	GTG/GTG/GTG/GTG	TAA/TAA/TAA/TAA		H
tRNA^Ser(UCN)^	7045/7048/7043/7043	7115/7118/7113/7113	71/71/71/71					TGA/TGA/TGA/TGA	L
tRNA^Asp^	7119/7122/7117/7117	7190/7193/7188/7188	72/72/72/72	3/3/3/3				GTC/GTC/GTC/GTC	H
CoX2	7204/7209/7202/7202	7894/7899/7892/7892	691/691/691/691	13/15/13/13	230/230/230/230	ATG/ATG/ATG/ATG	T–/T–/T–/T–		H
tRNA^Lys^	7895/7900/7893/7893	7970/7975/7968/7968	76/76/76/76					TTT/TTT/TTT/TTT	H
ATP8	7972/7977/7970/7970	8136/8141/8134/8134	165/165/165/165	1/1/1/1	54/54/54/54	ATG/ATG/ATG/ATG	TAA/TAA/TAA/TAA		H
ATP6	8130/8135/8128/8128	8813/8818/8811/8811	684/684/684/684	-7/-7/-7/-7	227/227/227/227	ATG/ATG/ATG/ATG	TAA/TAA/TAA/TAA		H
CoX3	8813/8818/8811/8811	9596/9601/9594/9594	784/784/784/784	-1/-1/-1/-1	261/261/261/261	ATG/ATG/ATG/ATG	T–/T–/T–/T–		H
tRNA^Gly^	9597/9602/9595/9595	9667/9673/9666/9666	71/72/72/72					TCC/TCC/TCC/TCC	H
NAD3	9668/9674/9667/9667	10017/10023/10016/10016	350/350/350/350		116/116/116/116	ATG/ATG/ATG/ATG	TA-/TA-/TA-/TA-		H
tRNA^Arg^	10018/10024/10017/10017	10087/10093/10085/10086	70/70/69/70					TCG/TCG/TCG/TCG	H
NAD4L	10088/10094/10086/10087	10384/10390/10382/10383	297/297/297/297		98/98/98/98	ATG/ATG/ATG/ATG	TAA/TAA/TAA/TAA		H
NAD4	10378/10384/10376/10377	11759/11765/11757/11758	1382/1382/1382/1382	-7/-7/-7/-7	460/459/460/460	ATG/ATG/ATG/ATG	TA-/TA-/TA-/TAA		H
tRNA^His^	11760/11766/11758/11759	11828/11834/11826/11827	69/69/69/69					GTG/GTG/GTG/GTG	H
tRNA^Ser(AGY )^	11829/11835/11827/11828	11897/11903/11895/11896	69/69/69/69					GCT/GCT/GCT/GCT	H
tRNA^Leu(CUN)^	11899/11905/11897/11898	11971/11977/11969/11970	73/73/73/73	1/1/1/1				TAG/TAG/TAG/TAG	H
NAD5	11972/11978/11970/11971	13807/13813/13805/13806	1836/1836/1836/1836		611/611/611/611	ATGATGATGATG	TAA/TAA/TAG/TAG		H
NAD6	13804/13810/13082/13803	14325/14331/14323/14324	522/522/522/522	-4/-4/-4/-4	173/173/173/174	ATGATGATGATG	TAA/TAA/TAG/TAG		L
tRNA^Glu^	14326/14332/14324/14325	14394/14400/14392/14393	69/69/69/69					TTC/TTC/TTC/TTC	L
Cytb	14397/14403/14397/14398	15537/15543/15537/15538	1141/1141/1141/1141	2/2/4/4	380/380/380/380	ATGATGATGATG	T–/T–/T–/T–		H
tRNA^Thr^	15538/15544/15538/15539	15609/15615/15608/15610	72/72/71/72					TGT/TGT/TGT/TGT	H
tRNA^Pro^	15609/15615/15608/15610	15678/15684/15677/15679	70/70/70/70	-1/-1/-1/-1				TGG/TGG/TGG/TGG	L
D-loop	15679/15685/15678/15680	16604/16609/16603/16603	926/925/926/924						H

**Table 3:** Nucleotide composition of the four newly determined Gobioninae mitochondrial genomes. The statistical results of the newly determined mitogenomes of *G. rivuloides*, *R. nasutus*, *M. chinssuensis*, and *M. elongate*.

	Size	AT content	Atskew	Gcskew
Genome	16604/16609/16603/16603	55.4/57.9/56.7/56.4	0.037/0.077/0.066/0.07	−0.176/ −0.243/ −0.217/ −0.215
PCGs	11414/11415/11414/11415	54.96/58.13/56.68/56.27	−0.0470.001 −0.008 −0.002	−0.19/ −0.262/ −0.239/ −0.238
tRNA	1563/1565/1561/1563	55.79/56.55/55.61/55.6	0.023/0.031/0.028/0.033	0.059/0.065/0.053/0.052
rRNA	2652/2653/2649/2649	53.66/54.69/54.17/54.28	0.249/0.272/0.247/0.248	−0.051/ −0.092/ −0.066/ −0.049

As reported in other teleost fishes, there were overlaps and gaps in the adjacent genes of mitogenomes (Satoh et al., 2016). Six gene overlapping regions were identified in the new mitogenomes, three of which exceeded four bases: tRNA^Ile^–tRNA^Gln^, ATP8–ATP6 (seven bases), ATP6–COX2, NAD4L–NAD4 (seven bases), NAD5–NAD6 (4 bases), and tRNA^Thr^-tRNA^Pro^ (Table 2). Compared with the published mitogenomes of 85 Gobioninae species, we found that the overlap of ATP8/ATP6, NAD4L/NAD4 and NAD5/NAD6 is common, with variation in the number of overlapping sequences ranging from five to eight (Table S4). The existence of gene overlaps can promote the miniaturization of the mitogenome and shorten the time of genome replication, which has potential kinetic advantages during the process of replication (Boyce, Zwick & Aquadro, 1990). In contrast to the overlapping characteristics, the locations and nucleotides of the intergenic spacers show greater diversity. In addition to the OL and control region, 13 intergenic spacers were recognized in the newly sequenced mitogenomes, ranging from 32 intergenic nucleotides in *G. rivuloides* to 34 intergenic nucleotides in *R. nasutus*, *M. elongata* and *M. chinssuensis* (Table 2)*.* The comparative analysis exhibited a greater diversity of intergenic spacers in Gobioninae species. In the intergenic spacer between tRNA^Asp^ and COX2, *Rhinogobio* was detected to have the longest intergenic nucleotides (15 bp). The genus *Sarcocheilichthys* had the largest variation, ranging from 6 bp (*S. parvus*) to 12 bp (*S. sinensis* and *S. lacustris*) (Table S4). The variety of overlaps and intergenic spacers between adjacent genes control the lengths of the mitogenomes (Huang & Liu, 2010).

### Protein-coding genes

The entire lengths of the 13 PCGs of the four sequenced mitogenomes were 11,414 bp for *G. rivuloides* and *M. elongata* and 11,415 bp for *M. chinssuensis* and *R. nasutus*. Twelve PCGs were located on the H-strand, while NAD6 was encoded on the L-strand. The total A + T content of the 13 PCGs varied from 55% in *G. rivuloides* to 58.1% in *R. nasutus* (Table 3). Unlike the whole-genome sequence, there was no obvious AT skew in the protein-coding genes. To better understand the degree of base bias of all PCGs, we calculated the AT skew and GC skew of each gene (Table 3). Twelve genes showed a negative GC skew, but NAD6 showed a positive CG, which is consistent with most teleost fish (Reyes et al., 1998; Gibson et al., 2005). We also calculated the nucleotide composition at each codon position of the 13 concatenated PCGs in Gobioninae species. The analysis showed that the second and third codon positions were much higher in AT content than the first codon positions, and the second codon positions presented a negative AT skew ranging from −0.37 to −0.38 (Table S5).

**Figure 2:**
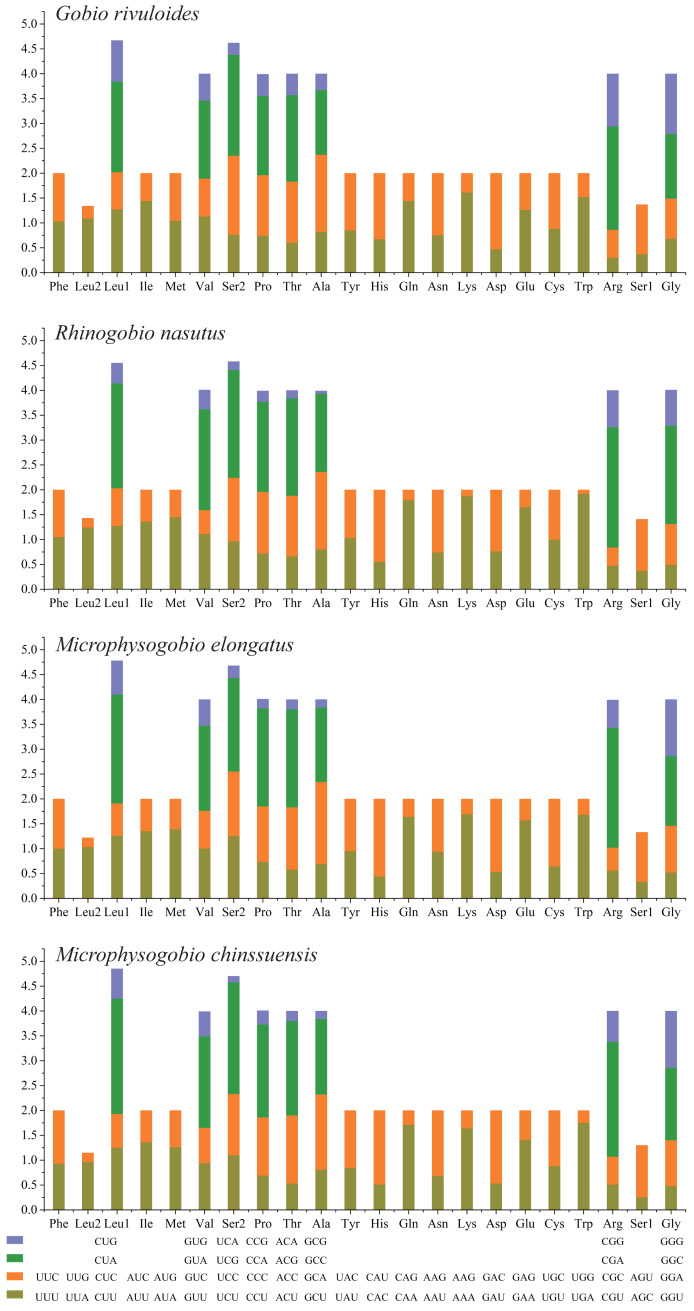
The relative synonymous codon usage (RSCU) of four Gobioninae mitochondrial genomes. The codons are shown on the *X*-axis and the RSCU values are shown on the *Y*-axis.

**Figure 3:**
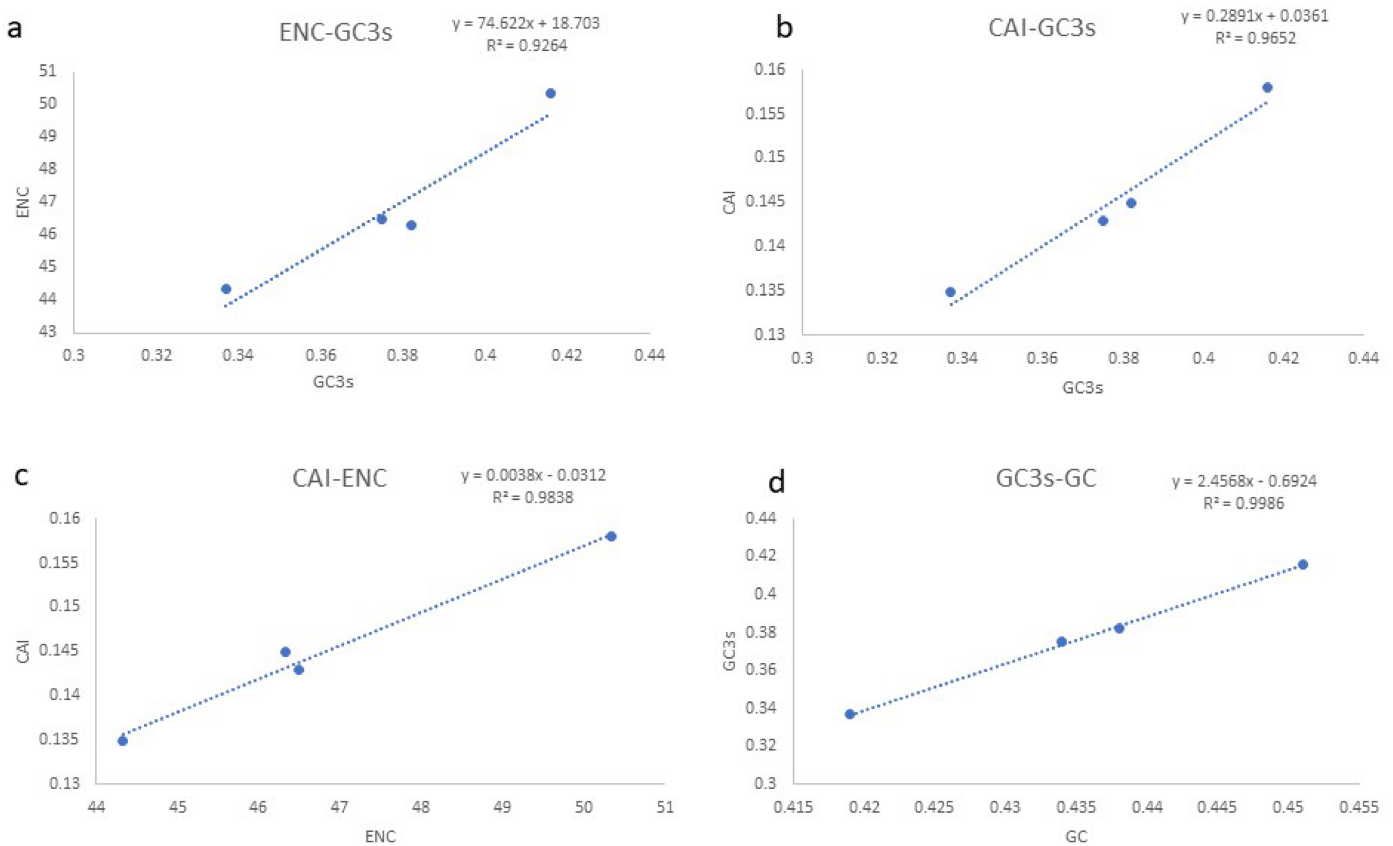
(A–D) Evaluation of codon bias across four Gobioninae mitochondrial genomes. ENC, effective number of codons (out of a maximum of 61); CAI, Codon adaption index; G+C, GC content of codons; (G+C)3, GC content of third codon positions.

Based on the comparative analysis, we found that most PCGs of Gobioninae species use the conventional ATN as the start codon, except for COX1 by GTG. However, the use of the termination codon in Gobioninae fishes appears to be diverse. The canonical termination codons (TAA and TAG) are used in most PCGs, whereas six PCGs utilized the incomplete termination codon (TA and T) (Table S6). Incomplete termination codons are commonly recognized across fish mitogenomes, and this may be related to posttranscriptional modification during the mRNA maturation process (Yu et al., 2019). To understand the genetic codon bias of the newly sequenced mitogenomes, we measured the RSCU, ENC, CAI and GC content of the third codon positions to evaluate codon usage bias. The RSCU results are summarized in Fig. 2 and Table S7. Leu (12.45–13.16%), Ala (8.56–8.98%), Thr (7.61–7.98%), and Ile (7.01–7.77%) were frequently encoded in the mitogenomes, and CUA, GCC, ACA, and AUU were used very regularly. In addition, an A- or T-ending codon occupies absolute dominance (Table S7), indicating a strong bias toward A and T, which is consistent with previous studies of Gobioninae fishes. The ENC was used to detect the codon usage bias of a single gene. Its variation ranged from 20 to 61 and reflected the preference degree of gene codon usage. A smaller Nc value indicates absolute bias toward a synonymous codon; in contrast, a higher Nc value reflects neutral codon usage (Wright, 1990). In the four sequenced species, the Nc values ranged from 36–51.2, indicating some trends of codon usage bias. CAI is commonly used to evaluate gene expression levels. A higher CAI value indicates a high gene expression level and a more significant codon bias (Sharp & Li, 1987). In detail, atp8 and three cytochrome oxidase subunits showed higher codon usage bias and codon expression levels, while atp6 exhibited lower gene expression levels than other protein-coding genes (Table S7). We also calculated the relationships of GC3s, ENC and CAI, and a positive correlation was observed between ENC and GC contents of the third codon positions (*R*^2^ = 0.93) (Fig. 3A) and CAI (*R*^2^ = 0.98) (Fig. 3B). The results show that the usage of codons in the newly sequenced mitogenomes was affected not only by GC content but also by gene expression level, which is consistent with previous studies (Duret & Mouchiroud, 1999; Berg, 1996; Broughton, Milam & Roe, 2001; Masta & Boore, 2004; Lee Woo-Jai, Huntting Howell & Kocher Thomas, 1995).

**Figure 4:**
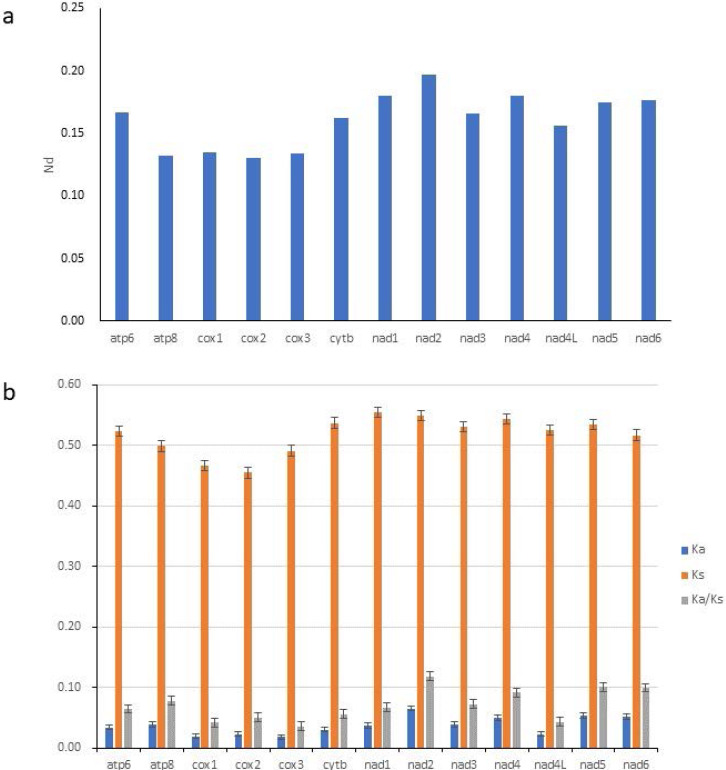
Nucleotide diversity (Nd, A) and the ratio of Ka/Ks (B) of PCGs inferred from 89 reported Gobioninae mitochondrial genomes.

To study the evolutionary rates of 13 PCGs in Gobioninae species, nucleotide diversity, Ka, Ks, and the average ratio of Ka/Ks (*ω*) were calculated for each PCG. The *ω* values of all PCGs were less than 1, indicating that all PCGs are evolving under purifying selection (Fig. 4B). The Ka/Ks analyses indicate that nad2 has the highest *ω* value (Fig. 4B) and is subjected to the least selection pressure. The nucleotide diversity of nad2 was the highest among all genes (Fig. 4A). Unlike nad2, cox1 and cox3 show lower *ω* values and nucleotide diversity (Fig. 4). Cox1 and cox3 are relatively conserved among the mitochondrial protein-coding genes due to their slower evolution rate and higher selection pressure.

### Transfer and ribosomal RNA genes

**Figure 5:**
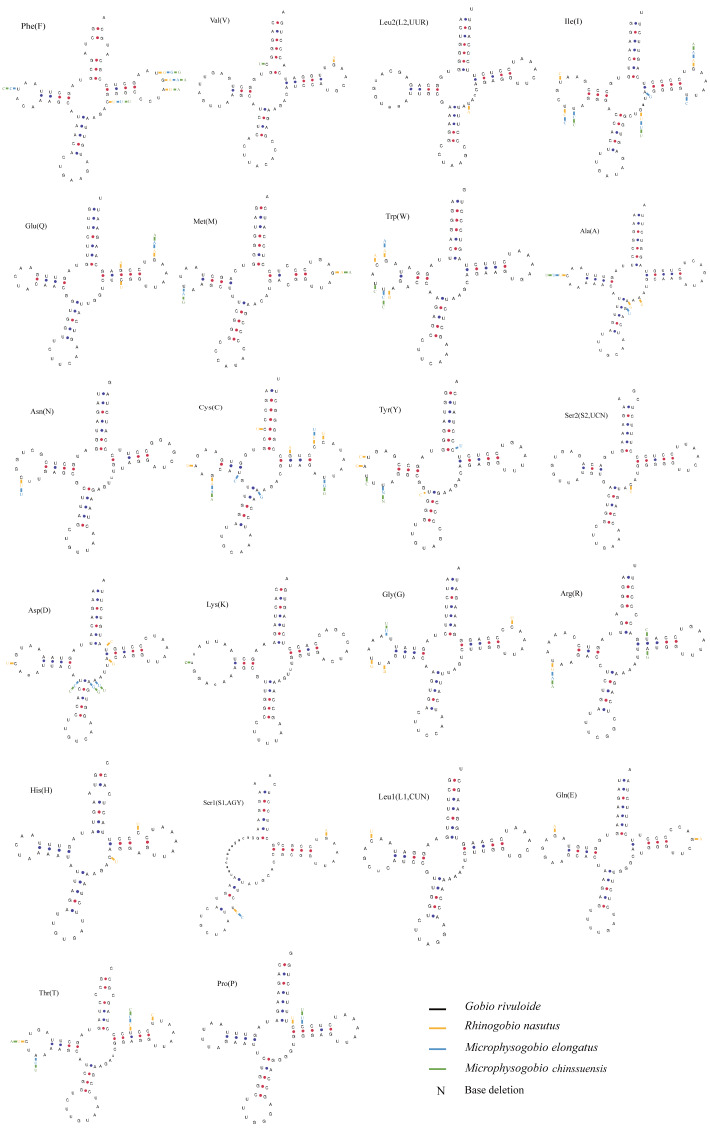
Putative secondary structures of tRNAs from the *Gobio rivuloide* mitogenome. The tRNAs are labeled with the abbreviations of their corresponding amino acids. The variable nucleotides of other three species compared with *Gobio rivuloides* were marked in different colour at corresponding sites.

The typical 22 tRNAs were detected in the four species and ranged from 68 bp (tRNA^Cys^) to 76 bp (tRNA^Lys^ and tRNA^Leu(UUR)^) in length. Among them, eight tRNAs were located on the L-strand, and the remaining 14 were located on the H-strand (Table 2). Most tRNAs can be folded into a typical cloverleaf secondary structure, except for tRNA^Ser^ (GCT) (L-strand), which lacks a dihydrouracil (DHU) arm (Fig. 5). The absence of DHU arms leads to incomplete secondary structures, which also occurs in the mitogenomes of other vertebrate fishes (Hwang, Byeon & Lee, 2014; Broughton, Milam & Roe, 2001). To be functional like normal tRNAs, these aberrant tRNAs may require coevolved interacting factors or posttranscriptional RNA editing (Masta & Boore, 2004). We also found that nucleotide substitutions occurred less frequently on tRNA stems than on loops through comparison of the secondary structures among the four sequenced mitogenomes (Fig. 5).

Two rRNA genes (12S rRNA and 16S rRNA) were recognized in newly sequenced mitogenomes. Among them, the larger ribosomal gene (16S) ranged from 1690 bp in *M. chinssuensis* to 1693 bp in *G. rivuloides* and *R. nasutus* and was located between tRNA^V al^ and tRNA^Leu(UUR)^. In contrast, the smaller ribosomal gene (12S) ranged from 958 bp in *M. elongata* to 960 bp in *R. nasutus* and was located between tRNA^Phe^ and tRNA^V al^ (Table 2).

### Noncoding regions

The origin of the light-strand replication region (OL) of the four new mitogenomes was located between tRNA^Asn^ and tRNA^Cys^ in the WANCY (tRNA^Trp^-tRNA^Ala^-tRNA^Asn^-tRNA^Cys^-tRNA^Tyr^) region and was between 30 bp (*G. rivuloides*) or 31 bp (*M. elongata*, *M. chinensis* and *R. nasutus*) in length. This sequence has palindrome characteristics and can be folded into a stable hairpin secondary structure (Fig. S1). Comparative analysis showed that the variation in OL that mainly occurred in the loops and stems was relatively conserved.

Similar to other teleost fish, the noncoding regions of the four newly sequenced mitogenomes were located between tRNA^Pro^ and tRNA^Phe^, with lengths ranging from 924 bp to 926 bp. In this region, we recognized the termination-associated sequence (TAS) and six conserved sequence blocks (CSB-D, CSB-E, CSB-F, CBS-1, CBS-2, and CBS-3) that are related to replication and transcription. The variations in the TAS sequence in the four measured mitochondria were great, except that the central ATGTATTATCACCAT remained consistent. In addition, the CSB-E and CSB-1 sequences of the four mitogenomes were identical and demonstrated a high degree of conservatism in this region. Although tandem repeat elements have been found in the control region of other fishes (Lee Woo-Jai, Huntting Howell & Kocher Thomas, 1995), we did not find them in the control regions of the four sequenced species.

### Phylogenetic analysis

**Figure 6:**
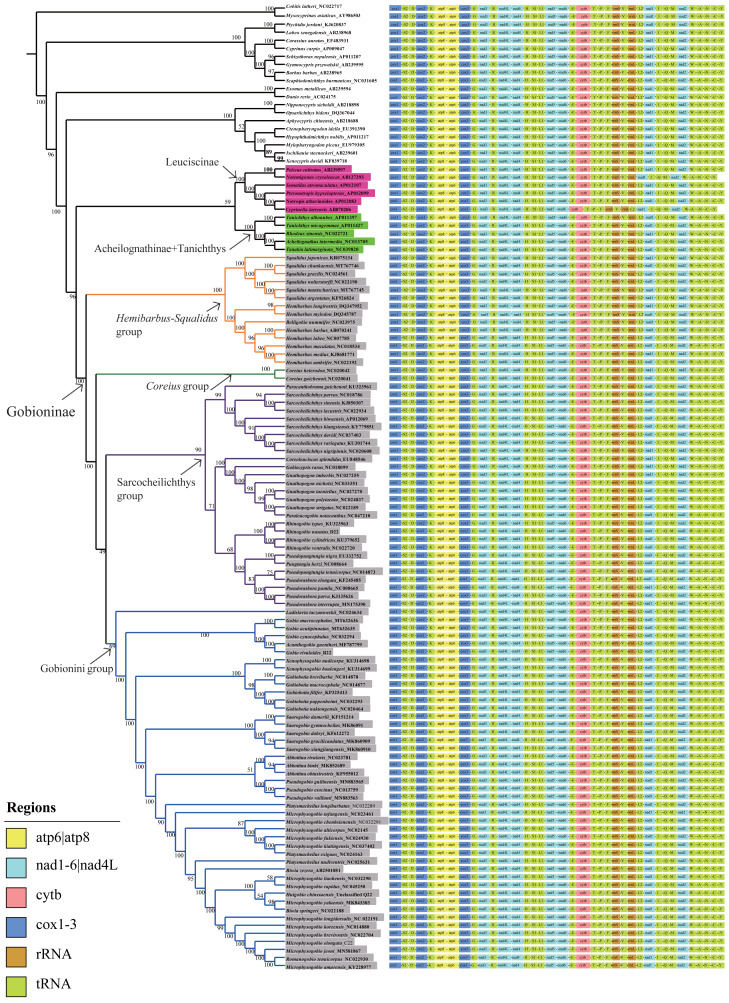
ML trees inferred from RaxML analyses based on PCGs+rRNAs database. Bootstrap support was assessed using 1,000 ultrafast bootstrap replicates and labelled on the nodes.

Phylogenetic analyses were performed on two datasets based on the same inference method (BI and ML) and yielded inferences regarding genus-level relationships. The relationships of Gobioninae obtained from the ML and BI analyses were similar (Fig. 6, Figs. S2–S4). An analysis of four phylogenetic trees (PP = 100, BI = 1) supports the monophyly of the subfamily Gobioninae. This conclusion is consistent with previous studies (Tang et al., 2011; Zhao et al., 2016). However, several studies have presented differing views on the phylogenetic position of Gobioninae. In some studies, Leuciscinae has been proposed as a sister group of Gobioninae (Tao, Mayden & He, 2013; Mayden & Chen, 2010). Some studies have shown that Gobioninae and Acheilognathinae or *Tanichthys* constitute sister groups (Tang et al., 2011; Wang, Li & He, 2007; Jiang et al., 2018). In this study, Gobioninae was a sister group of Leuciscinae + (Acheilognathinae + *Tanichthys*), as inferred from ML results. This is not completely consistent with previous studies (Wang, Li & He, 2007; Jiang et al., 2018).

Within Gobioninae, four major lineages were identified with strong support values. This lineage division is similar to the results of previous studies, but there are some discrepancies regarding the position of *Coreius*. In both ML and BI analysis, the *Hemibarbus-Squalidus* group consisted of *Hemibarbus*, *Belligobio,* and *Squalidus*. This group is thought to be a primitive group of Gobioninae, which was supported by both morphological characteristics and molecular data (Liu, Yang & Tang, 2010; Tang et al., 2011; Luo, Yue & Chen, 1977). In *Hemibarbus*-*Squalidus* clades, the genus *Squalidus* is monophyletic and is strongly supported with PP = 100 and BI = 1.00. The monophyly of *Hemibarbus* is rejected because of the presence of *Belligobio*.

In the ML analysis, *Pseudorasbora*, *Pseudopungtungia, Pungtungia*, *Rhinogobio*, *Gnathopogon*, *Paraleucogobio, Gobiocypris*, *Coreoleuciscus,* S*arcocheilichthys* and *Paracanthobrama* were placed in the *Sarcocheilichthys* group. In S*arcocheilichthys* clades, the monophyly of many genera is controversial except for *Paracanthobrama*, *Sarcocheilichthys*, *Coreoleuciscus* and *Rhinogobio*.

Based on all four trees, we can observe that *R. nasutus* is closely clustered with the species *Rhinogobio*. However, the monophyly of *Gnathopogon* is explicitly rejected, and the genus *Gnathopogon* is combined with *Gobiocypris* and *Paraleucogobio*, which agrees with the idea of *Paraleucogobio* being a junior synonym of *Gnathopogon* (Zhang & Fu, 2019). However, additional morphological evidence is needed to clarify the relationships between them. In addition, the taxonomic status of *Coreius* has been controversial. The genus *Coreius* is located at the base of Gobioninae and has strong support from the ML and BI trees in this study. It forms a sister group with species of *Sarcocheilichthys* and the Gobionini group. *Microphysogobio*, *Romanogobio*, *Biwia*, *Platysmacheilus*, *Abbottina*, *Pseudogobio*, S*aurogobio*, *Gobiobotia*, *Xenophysogobio*, *Acanthogobio*, *Gobio,* and *Ladislavia* were placed together and formed the Gobionini group. *Abbottina*, *Pseudogobio*, *Saurogobio*, *Gobiobotia*, *Xenophysogobio* and *Ladislavia* were obviously monophyletic.

However, the phylogenetic relationships of some other Gobionini species are problematic. Tang et al. (2011) indicated that *Ladislavia* was classified into the *Sarcocheilichthys* group, whereas the position of *Ladislavia* is at the base of the Gobionini group in this study. *Gobiobotia* and *Xenophysogobio* form a sister group that is placed in the subfamily Gobioninae rather than in Gobiobotinae. The position of *Pseudogobio* in previous research is contentious, while *Pseudogobio* and *Abbottina* comprise a sister group in our BI and ML analyses. The newly sequenced *G. rivuloides* and *Gobio* clustered together, while *Gobio* could not form a monophyletic group due to the existence of *Acanthogobio*.

The phylogenetic relationships among *Microphysogobio*, *Romanogobio*, *Hugobio*, *Biwia* and *Platysmacheilus* require further study. These variable phylogenetic relationships may be influenced by the following aspects: (1) different data (single genes or multigenes of mitogenomes) and different analysis methods, (2) specimen availability of different species or lineages used in the studies (Yang et al., 2006; Liu, Yang & Tang, 2010; Tang et al., 2011; Zhao et al., 2016; Kim et al., 2013), and (3) the evolutionary characteristics of mitogenomes (Feng et al., 2022; Liu et al., 2022). According to recent studies, the robust phylogenetic relationship may be affected by introgression, hybridization, and incomplete lineage sorting (Feng et al., 2022; Zou et al., 2022; Liu et al., 2022). Hence, addressing the complex phylogenetic relationships of Gobionini lineages requires comprehensive samples from all genera, more mitogenomes and informative variable sites in the genome.

## Conclusion

The mitogenomes of four Gobioninae species (*Microphysogobio elongatus*, *Microphysogobio chinssuensis*, *Gobio rivuloides* and *Rhinogobio nasutus*) were newly sequenced and analyzed in this study. Structure and evolutionary analyses of the mitogenomes of Gobioninae were conducted. The results showed that these four mitogenomes were biased toward A/T, and NAD4 was subjected to low purification selection and had a faster evolution rate among the 13 PCGs. Phylogenetic analysis indicated that the four species clustered together with their congener species. However, further study is needed to investigate the phylogenetic relationships among *Microphysogobio*, *Romanogobio*, *Hugobio*, *Biwia* and *Platysmacheilus.* Evolving more comprehensive samples from all genera and incorporating more informative variable sites in the genome will be an effective method.

##  Supplemental Information

10.7717/peerj.16632/supp-1 Supplemental Information 1 Four new mitochondrial genomes WANCY region sequences have palindrome characteristics and can be folded into a stable hairpin secondary structure

10.7717/peerj.16632/supp-2 Supplemental Information 2 ML trees inferred from RaxML analyses based on the database of PCGs; bootstrap support was assessed using 1000 ultrafast bootstrap replicates and labelled on the nodes

10.7717/peerj.16632/supp-3 Supplemental Information 3 BI trees inferred from MrBayes analyses based on the database of PCGs and rRNAs (10 million generations, 25% burn-in, 50% majority-rule consensus trees; branch support was assessed using posterior probabilities (PP))

10.7717/peerj.16632/supp-4 Supplemental Information 4 BI trees inferred from MrBayes analyses based on the database of PCGs (10 million generations, 25% burn-in, 50% majority-rule consensus trees; branch support was assessed using posterior probabilities (PP))

10.7717/peerj.16632/supp-5 Supplemental Information 5 Detailed information about the mitochondrial genomes of the species in this study

10.7717/peerj.16632/supp-6 Supplemental Information 6 Primer sequences used this study in the PCR reaction

10.7717/peerj.16632/supp-7 Supplemental Information 7 Different partition schemes of the PCGs in the phylogenetic analysis based on PartitionFinder software

10.7717/peerj.16632/supp-8 Supplemental Information 8 Positional relationships of genes/regions of the 89 mitochondrial genomes studied

10.7717/peerj.16632/supp-9 Supplemental Information 9 AT Skew and GC Skew of protein coding genes of the 89 mitochondrial genomes studied (The four newly sequenced mitochondrial genomes are highlighted in bold blue)

10.7717/peerj.16632/supp-10 Supplemental Information 10 Stop codons of the PCGs of the mitochondrial genomes studied

10.7717/peerj.16632/supp-11 Supplemental Information 11 Relative synonymous codon usage (RSCU) of four Gobioninae mitochondrial genomes studied

10.7717/peerj.16632/supp-12 Supplemental Information 12 Raw data and the assembled sequence

## Abbreviations

PCGs: protein coding genes
bp: base pairs
CAI: Codon adaptation index
RSCU: relative synonymous codon usage
Ka: nonsynonymous substitution
Ks: synonymous substitution
ENC: effective codon usage statistics
ML: maximum likelihood
BI: Bayesian inference
BS: bootstrap support
tRNAs: transfer RNA genes
rRNAs: ribosomal RNA genes
*ω*: the average ratio of Ka/Ks
OL: origin of the light-strand replication region
WANCY: (tRNA^Trp^-tRNA^Ala^-tRNA^Asn^-tRNA^Cys^-tRNA^Tyr^)
TAS: termination associated sequence
CSB: conserved sequence blocks
atp6 (APT6): ATP synthase F0 subunit 6
atp8 (ATP8): ATP synthase F0 subunit 8
cox1 (COI): cytochrome c oxidase subunit I
cox2 (COII): cytochrome c oxidase subunit II
cox3 (COIII): cytochrome c oxidase subunit III
Cytb: cytochrome b
nad1 (NAD1): NADH dehydrogenase subuint 1
nad2 (NAD2): NADH dehydrogenase subuint 2
nad3 (NAD3): NADH dehydrogenase subuint 3
nad4 (NAD4): NADH dehydrogenase subuint 4
nad4L (NAD4L): NADH dehydrogenase subuint 4 L
nad5 (NAD5): NADH dehydrogenase subuint 5
nad6 (NAD6): NADH dehydrogenase subuint 6
RAG1 (2): Recombination activating gene1 (2)
Rh: rhodopsin

## Additional Information and Declarations

### Data Availability

The following information was supplied regarding data availability: The sequences are available in the Supplemental Files and at GenBank: ON758294– ON758297.
