# Supplementary material for: Four new complete mitochondrial genomes of Gobioninae fishes (Teleostei: Cyprinidae) and their phylogenetic implications
Source: PeerJ. 2024 Jan 19;12:e16632. doi: 10.7717/peerj.16632 (PMC10802160; doi:10.7717/peerj.16632)
Supplement: Supplemental Information 1 [file peerj-12-16632-s001.pdf]

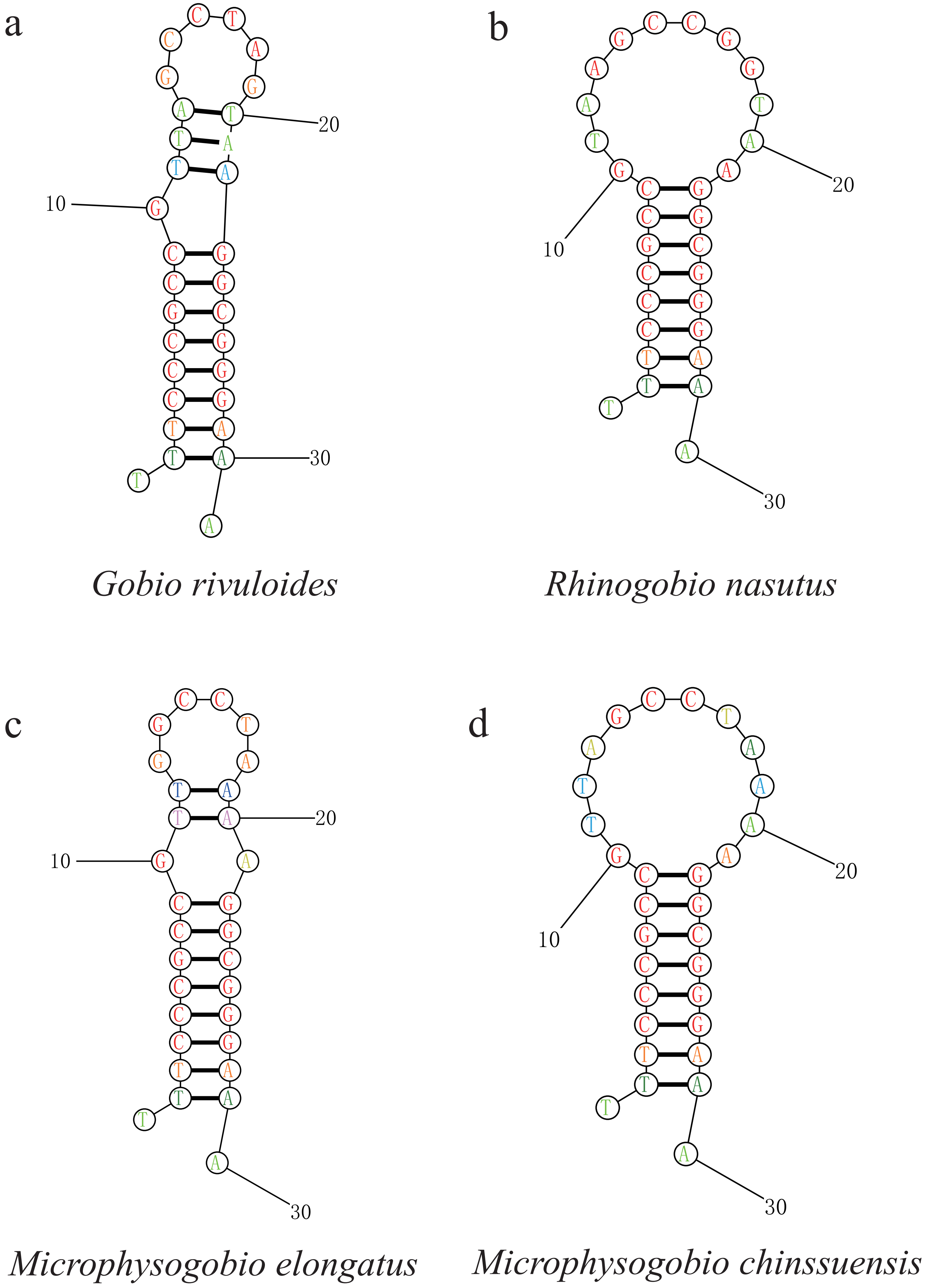
a
20
10
30
Gobio rivuloides
b
20
10
30
Rhinogobio nasutus
c
20
10
30
Microphysogobio elongatus
d
20
10
30
Microphysogobio chinssuensis
