# Supplementary figures and images for: Four new complete mitochondrial genomes of Gobioninae fishes (Teleostei: Cyprinidae) and their phylogenetic implications
Source: PeerJ. 2024 Jan 19;12:e16632. doi: 10.7717/peerj.16632 (PMC10802160; doi:10.7717/peerj.16632)

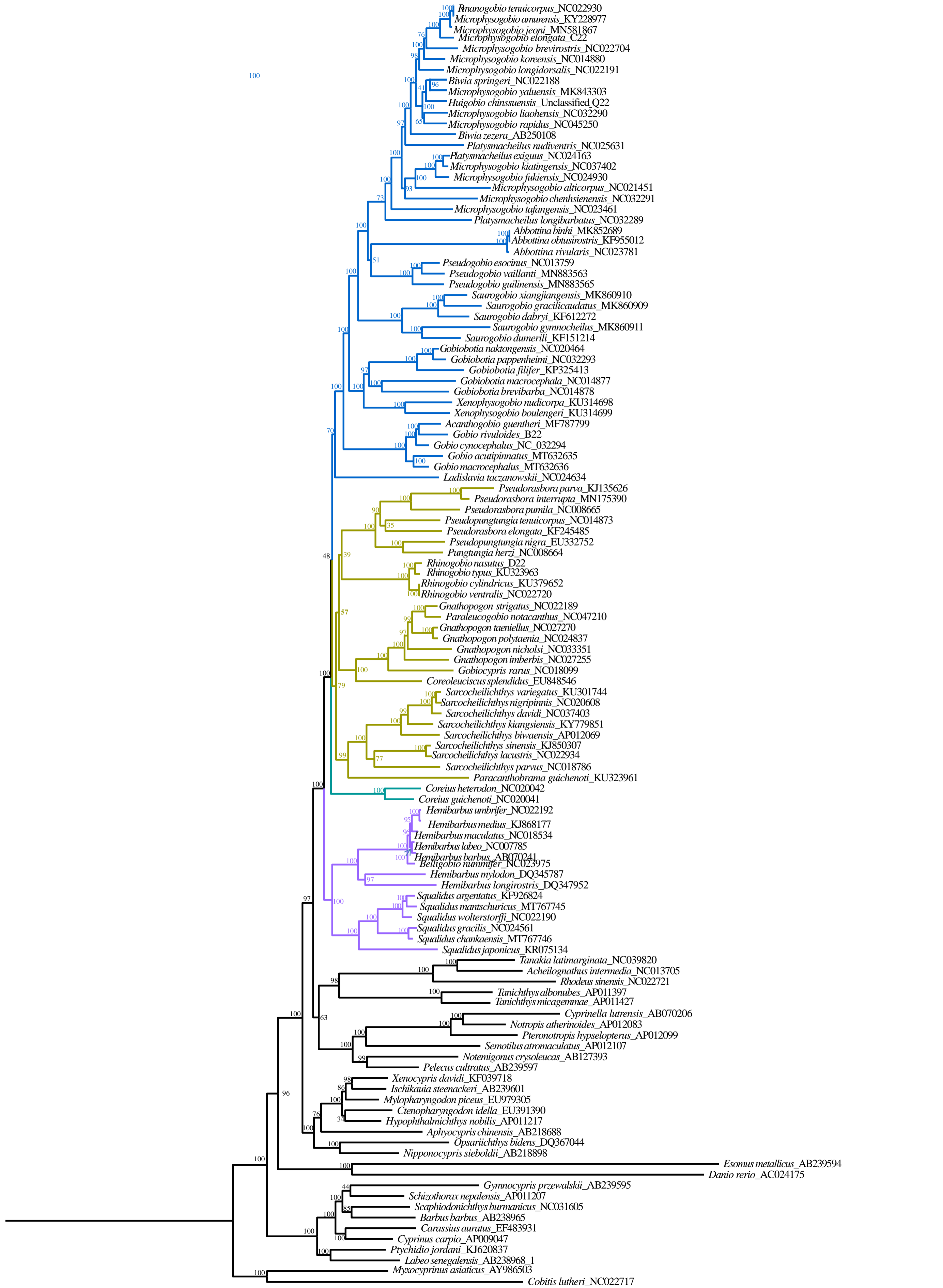

0.3

Supplement: Supplemental Information 2 [file peerj-12-16632-s002.pdf]

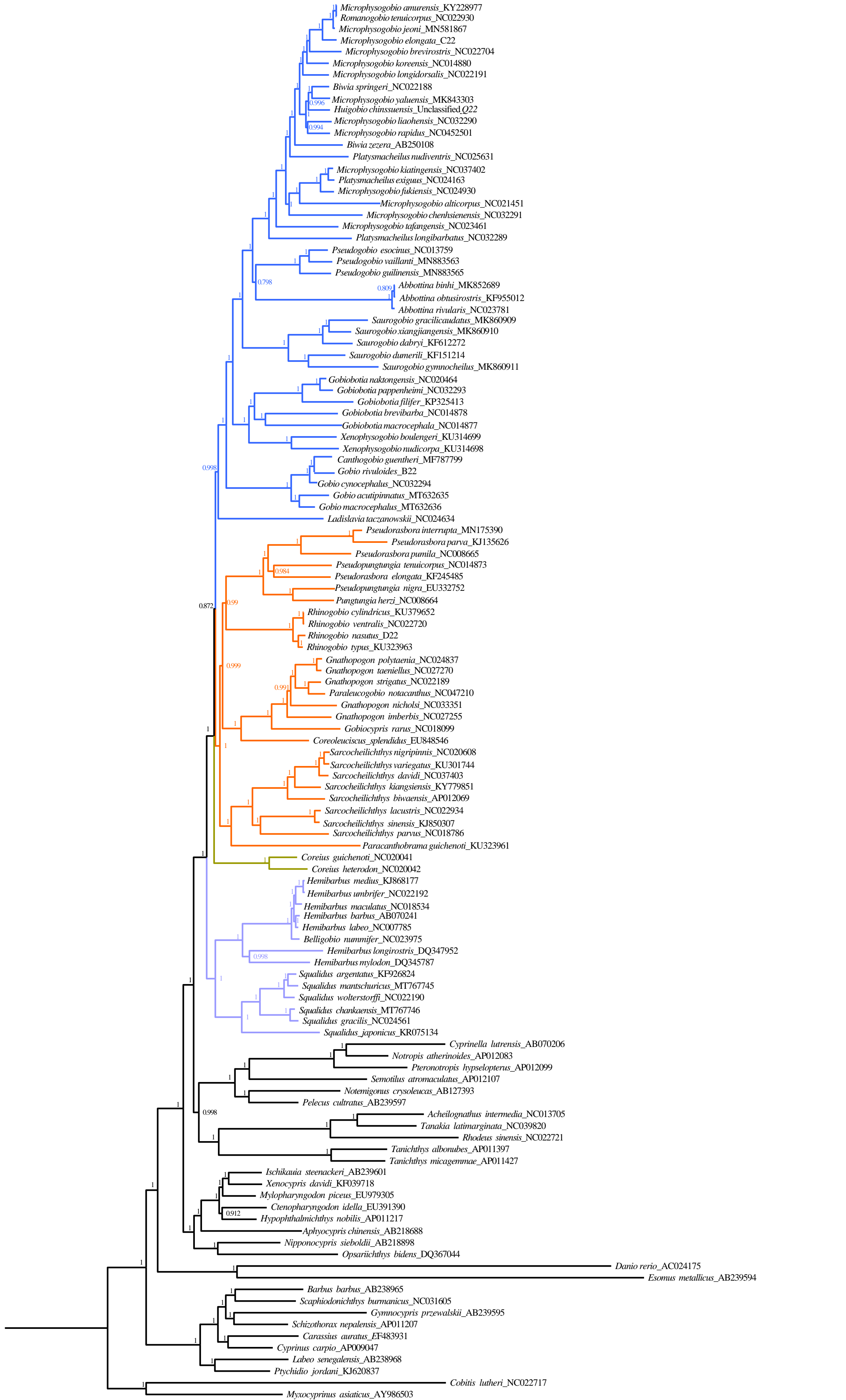

Supplement: Supplemental Information 3 [file peerj-12-16632-s003.pdf]

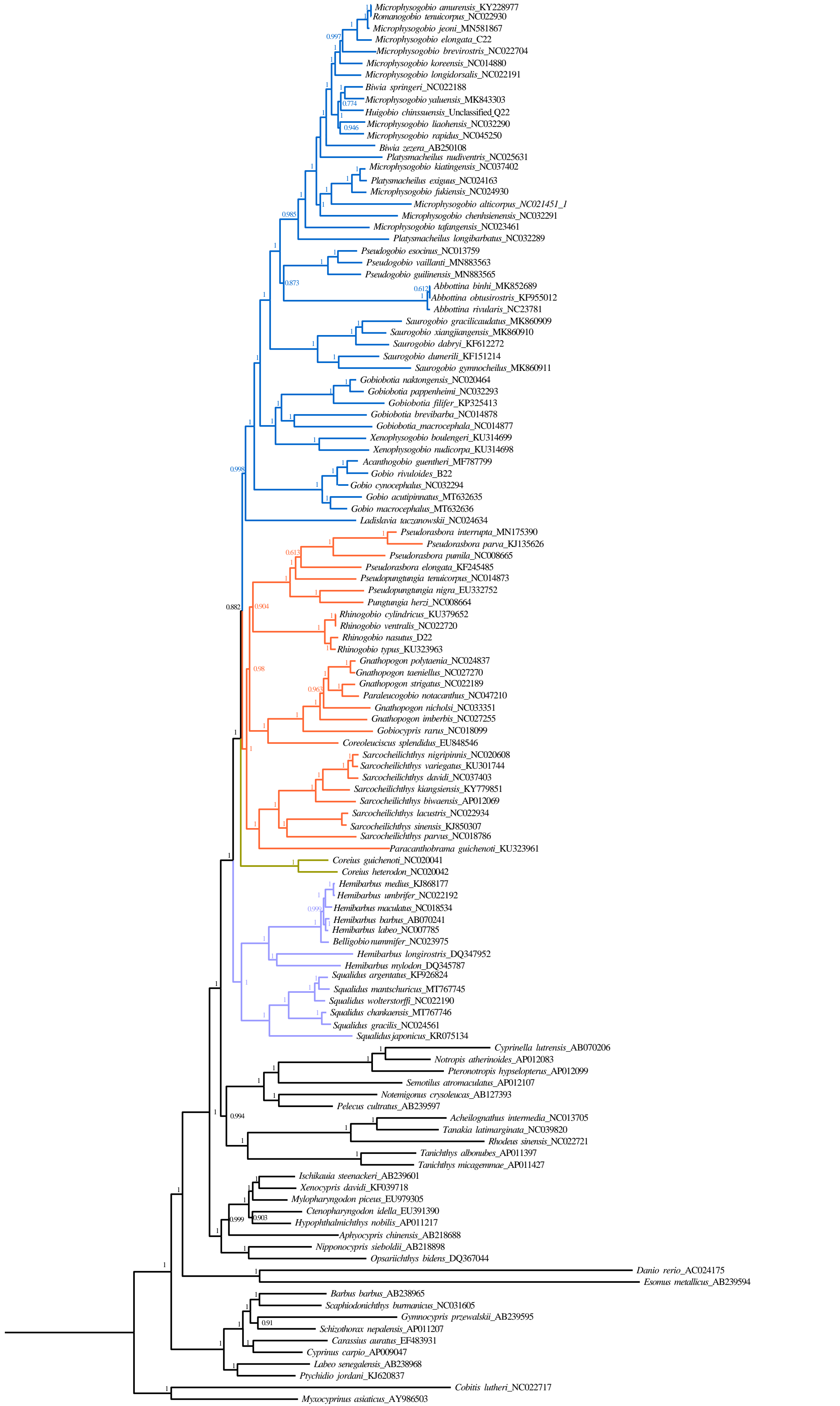

Supplement: Supplemental Information 4 [file peerj-12-16632-s004.pdf]
