# Supplementary material for: Four new complete mitochondrial genomes of Gobioninae fishes (Teleostei: Cyprinidae) and their phylogenetic implications
Source: PeerJ. 2024 Jan 19;12:e16632. doi: 10.7717/peerj.16632 (PMC10802160; doi:10.7717/peerj.16632)
Supplement: Supplemental Information 5 [file peerj-12-16632-s005.doc]

TABLE S1 Information of the sample in this study.

| Accession Number | Species Name | Subfamily | Family | Length | AT% |
| --- | --- | --- | --- | --- | --- |
| AB070206.1 | *Cyprinella lutrensis* | Pogonichthyinae | Leuciscidae | 16706 | 54.9 |
| AB070241.1 | *Hemibarbus barbus* | Gobioninae | Cyprinidae | 16681 | 55.5 |
| AB127393.1 | *Notemigonus crysoleucas* | Leuciscinae | Cyprinidae | 16583 | 55.8 |
| AB218688.1 | *Aphyocypris chinensis* | Xenocypridinae | Cyprinidae | 16606 | 58.4 |
| AB218898.1 | *Nipponocypris sieboldii* | Xenocypridinae | Xenocyprididae | 16616 | 55.9 |
| AB238965.1 | *Barbus barbus* | Barbinae | Cyprinidae | 16600 | 56.4 |
| AB238968.1 | *Labeo senegalensis* | Labeoninae | Cyprinidae | 16604 | 57 |
| AB239594.1 | *Esomus metallicus* | Danioninae | Cyprinidae | 17029 | 59.7 |
| AB239595.1 | *Gymnocypris przewalskii* | Schizopygopsinae | Cyprinidae | 16682 | 56 |
| AB239597.1 | *Pelecus cultratus* | Leuciscinae | Cyprinidae | 16610 | 57.3 |
| AB239601.1 | *Ischikauia steenackeri* | Xenocypridinae | Xenocyprididae | 16620 | 56.5 |
| AB250108.1 | *Biwia zezera* | Gobioninae | Cyprinidae | 16599 | 57.1 |
| AC024175.3 | *Danio rerio* | Danioninae | Cyprinidae | 16596 | 60 |
| AP009047.1 | *Cyprinus carpio* | Cypriniae | Cyprinidae | 16580 | 56.7 |
| AP011207.1 | *Schizothorax nepalensis* | Schizothoracinae | Cyprinidae | 16589 | 55.4 |
| AP011217.1 | *Hypophthalmichthys nobilis* | Xenocypridinae | Xenocyprididae | 16614 | 56.9 |
| AP011397.1 | *Tanichthys albonubes* | Tanichthyidae | Cyprinidae | 16547 | 60.3 |
| AP011427.1 | *Tanichthys micagemmae* | Tanichthyidae | Cyprinidae | 16555 | 60.9 |
| AP012069.1 | *Sarcocheilichthys biwaensis* | Gobioninae | Cyprinidae | 16665 | 54.8 |
| AP012083.1 | *Notropis atherinoides* | Pogonichthyinae | Leuciscidae | 16710 | 54.2 |
| AP012099.1 | *Pteronotropis hypselopterus* | Pogonichthyinae | Leuciscidae | 16710 | 57.9 |
| AP012107.1 | *Semotilus atromaculatus* | Plagopterinae | Leuciscidae | 16623 | 56.3 |
| AY986503.1 | *Myxocyprinus asiaticus* | Myxocyprininae | Catostomidae | 16623 | 53.3 |
| B22 | *Gobio rivuloides* | Gobioninae | Cyprinidae | 16604 | 55.4 |
| C22 | *Microphysogobio elongata* | Gobioninae | Cyprinidae | 16603 | 56.7 |
| D22 | *Rhinogobio nasutus* | Gobioninae | Cyprinidae | 16609 | 57.9 |
| DQ345787.2 | *Hemibarbus mylodon* | Gobioninae | Cyprinidae | 16605 | 55.7 |
| DQ347952.2 | *Hemibarbus longirostris* | Gobioninae | Cyprinidae | 16608 | 54 |
| DQ367044.1 | *Opsariichthys bidens* | Xenocypridinae | Xenocyprididae | 16611 | 53.9 |
| EF483931.1 | *Carassius auratus* | Cypriniae | Cyprinidae | 16581 | 57.6 |
| EU332752.1 | *Pseudopungtungia nigra* | Gobioninae | Cyprinidae | 16605 | 57.5 |
| EU391390.1 | *Ctenopharyngodon idella* | Xenocypridinae | Cyprinidae | 16609 | 58.1 |
| EU848546.1 | *Coreoleuciscus splendidus* | Gobioninae | Cyprinidae | 16566 | 58.2 |
| EU979305.1 | *Mylopharyngodon piceus* | Xenocypridinae | Xenocyprididae | 16609 | 56.5 |
| KF039718.1 | *Xenocypris davidi* | Xenocypridinae | Xenocyprididae | 16630 | 56.7 |
| KF151214.1 | *Saurogobio dumerili* | Gobioninae | Cyprinidae | 16601 | 55.4 |
| KF245485.1 | *Pseudorasbora elongata* | Gobioninae | Cyprinidae | 16607 | 59.5 |
| KF612272.1 | *Saurogobio dabryi* | Gobioninae | Cyprinidae | 16601 | 56.1 |
| KF926824.1 | *Squalidus argentatus* | Gobioninae | Cyprinidae | 16607 | 55.9 |
| KF955012.1 | *Abbottina obtusirostris* | Gobioninae | Cyprinidae | 16605 | 55.8 |
| KJ135626.1 | *Pseudorasbora parva* | Gobioninae | Cyprinidae | 16601 | 58.5 |
| KJ620837.1 | *Ptychidio jordani* | Labeoninae | Cyprinidae | 16602 | 57.6 |
| KJ850307.1 | *Sarcocheilichthys sinensis* | Gobioninae | Cyprinidae | 16683 | 56.8 |
| KJ868177.1 | *Hemibarbus medius* | Gobioninae | Cyprinidae | 16611 | 55.7 |
| KP325413.1 | *Gobiobotia filifer* | Gobioninae | Cyprinidae | 16613 | 55.3 |
| KR075134.1 | *Squalidus japonicus* | Gobioninae | Cyprinidae | 16597 | 52.6 |
| KU301744.1 | *Sarcocheilichthys variegatus* | Gobioninae | Cyprinidae | 16678 | 56.6 |
| KU314698.1 | *Xenophysogobio nudicorpa* | Gobioninae | Cyprinidae | 16617 | 57.1 |
| KU314699.1 | *Xenophysogobio boulengeri* | Gobioninae | Cyprinidae | 16615 | 57.8 |
| KU323961.1 | *Paracanthobrama guichenoti* | Gobionidae | Cyprinidae | 16607 | 59.7 |
| KU323963.1 | *Rhinogobio typus* | Gobioninae | Cyprinidae | 16607 | 57.8 |
| KU379652.1 | *Rhinogobio cylindricus* | Gobioninae | Cyprinidae | 16607 | 57.8 |
| KY228977.1 | *Microphysogobio amurensis* | Gobioninae | Cyprinidae | 16605 | 56.1 |
| KY779851.1 | *Sarcocheilichthys kiangsiensis* | Gobioninae | Cyprinidae | 16672 | 55.4 |
| MF787799.1 | *Acanthogobio guentheri* | Gobioninae | Cyprinidae | 16604 | 55.5 |
| MK843303.1 | *Microphysogobio yaluensis* | Gobioninae | Cyprinidae | 16602 | 56.2 |
| MK852689.1 | *Abbottina binhi* | Gobioninae | Cyprinidae | 16599 | 55.6 |
| MK860909.1 | *Saurogobio gracilicaudatus* | Gobioninae | Cyprinidae | 16608 | 57 |
| MK860910.1 | *Saurogobio xiangjiangensis* | Gobioninae | Cyprinidae | 16600 | 56.2 |
| MK860911.1 | *Saurogobio gymnocheilus* | Gobioninae | Cyprinidae | 16604 | 55.9 |
| MN175390.1 | *Pseudorasbora interrupta* | Gobioninae | Cyprinidae | 16601 | 59 |
| MN581867.2 | *Microphysogobio jeoni* | Gobioninae | Cyprinidae | 16602 | 56.1 |
| MN883563.1 | *Pseudogobio vaillanti* | Gobioninae | Cyprinidae | 16605 | 56.7 |
| MN883565.1 | *Pseudogobio guilinensis* | Gobioninae | Cyprinidae | 16609 | 57.5 |
| MT632635.1 | *Gobio acutipinnatus* | Gobioninae | Cyprinidae | 16609 | 54.9 |
| MT632636.1 | *Gobio macrocephalus* | Gobioninae | Cyprinidae | 16609 | 56.6 |
| MT767745.1 | *Squalidus mantschuricus* | Gobioninae | Cyprinidae | 16605 | 56 |
| MT767746.1 | *Squalidus chankaensis* | Gobioninae | Cyprinidae | 16611 | 55.4 |
| NC_007785.2 | *Hemibarbus labeo* | Gobioninae | Cyprinidae | 16612 | 55.7 |
| NC_008664.1 | *Pungtungia herzi* | Gobioninae | Cyprinidae | 16600 | 57.7 |
| NC_008665.1 | *Pseudorasbora pumila* | Gobioninae | Cyprinidae | 16601 | 58.9 |
| NC_013705.1 | *Acheilognathus intermedia* | Acheilognathidae | Cyprinidae | 16610 | 54.5 |
| NC_013759.1 | *Pseudogobio esocinus* | Gobioninae | Cyprinidae | 16609 | 56.8 |
| NC_014873.1 | *Pseudopungtungia tenuicorpus* | Gobioninae | Cyprinidae | 16590 | 56.9 |
| NC_014877.1 | *Gobiobotia macrocephala* | Gobioninae | Cyprinidae | 16610 | 54.8 |
| NC_014878.1 | *Gobiobotia brevibarba* | Gobioninae | Cyprinidae | 16594 | 54.9 |
| NC_014880.1 | *Microphysogobio koreensis* | Gobioninae | Cyprinidae | 16606 | 56.2 |
| NC_018099.1 | *Gobiocypris rarus* | Gobioninae | Cyprinidae | 16601 | 57.1 |
| NC_018534.1 | *Hemibarbus maculatus* | Gobioninae | Cyprinidae | 16611 | 55.6 |
| NC_018786.1 | *Sarcocheilichthys parvus* | Gobioninae | Cyprinidae | 16677 | 56.4 |
| NC_020041.1 | *Coreius guichenoti* | Gobioninae | Cyprinidae | 16612 | 57.5 |
| NC_020042.1 | *Coreius heterodon* | Gobioninae | Cyprinidae | 16611 | 60.1 |
| NC_020464.1 | *Gobiobotia naktongensis* | Gobioninae | Cyprinidae | 16609 | 57 |
| NC_020608.1 | *Sarcocheilichthys nigripinnis* | Gobioninae | Cyprinidae | 16680 | 56.5 |
| NC_021451.1 | *Microphysogobio alticorpus* | Gobioninae | Cyprinidae | 16568 | 56.2 |
| NC_022189.1 | *Gnathopogon strigatus* | Gobioninae | Cyprinidae | 16602 | 56.7 |
| NC_022190.1 | *Squalidus wolterstorffi* | Gobioninae | Cyprinidae | 16602 | 56 |
| NC_022191.1 | *Microphysogobio longidorsalis* | Gobioninae | Cyprinidae | 16603 | 55.8 |
| NC_022192.1 | *Hemibarbus umbrifer* | Gobioninae | Cyprinidae | 16611 | 55.7 |
| NC_022704.1 | *Microphysogobio brevirostris* | Gobioninae | Cyprinidae | 16608 | 56.6 |
| NC_022717.1 | *Cobitis lutheri* | Cobitinae | Cobitidae | 16639 | 58.8 |
| NC_022720.1 | *Rhinogobio ventralis* | Gobioninae | Cyprinidae | 16607 | 57.7 |
| NC_022721.1 | *Rhodeus sinensis* | Acheilognathidae | Cyprinidae | 16677 | 56.3 |
| NC_022930.1 | *Romanogobio tenuicorpus* | Gobioninae | Cyprinidae | 16600 | 56.1 |
| NC_022934.1 | *Sarcocheilichthys lacustris* | Gobioninae | Cyprinidae | 16683 | 56.6 |
| NC_023461.1 | *Microphysogobio tafangensis* | Gobioninae | Cyprinidae | 16605 | 54.9 |
| NC_023781.1 | *Abbottina rivularis* | Gobioninae | Cyprinidae | 16597 | 55.7 |
| NC_024163.1 | *Platysmacheilus exiguus* | Gobioninae | Cyprinidae | 16604 | 56.9 |
| NC_024561.1 | *Squalidus gracilis* | Gobioninae | Cyprinidae | 16605 | 55.5 |
| NC_024634.1 | *Ladislavia taczanowskii* | Gobioninae | Cyprinidae | 16613 | 55.3 |
| NC_024837.1 | *Gnathopogon polytaenia* | Gobioninae | Cyprinidae | 16594 | 56.1 |
| NC_024930.1 | *Microphysogobio fukiensis* | Gobioninae | Cyprinidae | 16600 | 57 |
| NC_025631.1 | *Platysmacheilus nudiventris* | Gobioninae | Cyprinidae | 16603 | 57.1 |
| NC_027255.1 | *Gnathopogon imberbis* | Gobioninae | Cyprinidae | 16598 | 57 |
| NC_027270.1 | *Gnathopogon taeniellus* | Gobioninae | Cyprinidae | 16596 | 56.3 |
| NC_031605.1 | *Scaphiodonichthys burmanicus* | Barbinae | Cyprinidae | 16592 | 57.4 |
| NC_032289.1 | *Platysmacheilus longibarbatus* | Gobioninae | Cyprinidae | 16615 | 56.3 |
| NC_032290.1 | *Microphysogobio liaohensis* | Gobioninae | Cyprinidae | 16609 | 56.6 |
| NC_032291.1 | *Microphysogobio chenhsienensis* | Gobioninae | Cyprinidae | 16610 | 58 |
| NC_032293.1 | *Gobiobotia pappenheimi* | Gobioninae | Cyprinidae | 16605 | 56.8 |
| NC_032294.1 | *Gobio cynocephalus* | Gobioninae | Cyprinidae | 16605 | 56.1 |
| NC_033351.1 | *Gnathopogon nicholsi* | Gobioninae | Cyprinidae | 16606 | 56.9 |
| NC_037402.1 | *Microphysogobio kiatingensis* | Gobioninae | Cyprinidae | 16603 | 56.9 |
| NC_037403.1 | *Sarcocheilichthys davidi* | Gobioninae | Cyprinidae | 16675 | 56.1 |
| NC_039820.1 | *Tanakia latimarginata* | Acheilognathidae | Cyprinidae | 16568 | 54.5 |
| NC_045250.1 | *Microphysogobio rapidus* | Gobioninae | Cyprinidae | 16603 | 56.1 |
| NC_047210.1 | *Paraleucogobio notacanthus* | Gobioninae | Cyprinidae | 16596 | 56.4 |
| NC022188.1 | *Biwia springeri* | Gobioninae | Cyprinidae | 16606 | 56.7 |
| NC023975.1 | *Belligobio nummifer* | Gobioninae | Cyprinidae | 16610 | 55.8 |
| Q22-1 | *Microphysogobio chinssuensis* | Gobioninae | Cyprinidae | 16603 | 56.4 |
