# Supplementary material for: Four new complete mitochondrial genomes of Gobioninae fishes (Teleostei: Cyprinidae) and their phylogenetic implications
Source: PeerJ. 2024 Jan 19;12:e16632. doi: 10.7717/peerj.16632 (PMC10802160; doi:10.7717/peerj.16632)
Supplement: Supplemental Information 6 [file peerj-12-16632-s006.doc]

TABLE S2 Primers sequences used this study in the PCR reaction.

| **Primer name** | **Primer sequence(5'-3')** | **Location** | **Length (bp)** |
| --- | --- | --- | --- |
| G1F | CAGCAGTGATARACATTRAGCC | 237 | 22 |
| G1R | TCTTTCAGCKTTCCCTTGCG | 1242 | 20 |
| G2F | GGCGTGGCTGAGTTAGTT | 1031 | 18 |
| G2R | GTGTTTGCCGAGTTCCTT | 1989 | 18 |
| G3F | GCTAAAATGAGTAACAAGAAG | 1795 | 21 |
| G3R | GCCACCCCAGTATGCCYTTAT | 2783 | 21 |
| G4F | GYAATCCAGGTCAGTTTCT | 2633 | 19 |
| G4R | GCCTCCNGCRTAYTCTAC | 3524 | 18 |
| G5F | TCAGGDTGRGCATCAAAYTC | 3242 | 20 |
| G5R | GTATGGGCCCGAAAGCTTHATT | 4004 | 22 |
| G6F | TACCCACGATTCCGTTATG | 3716 | 19 |
| G6R | AATGCYGCBGAGGTYATG | 4687 | 18 |
| G7F | GCCTAYTCCTCYATYGC | 4597 | 17 |
| G7R | AAGCAGTYCCCACTATTCC | 5571 | 19 |
| G8F | TCAARCCAGCGAGCATCCAT | 5300 | 20 |
| G8R | CRTGTGAAATRATNCCAAAT | 6260 | 20 |
| G9F | GCCGCTGGAATTACAATA | 6097 | 17 |
| G9R | AGTTCTTCTATAACGGGTG | 7243 | 19 |
| G10F | ACGAGAAAGGRAGGAATTGAAC | 7043 | 22 |
| G10R | TTATCATGGTCARTYTCARG | 8116 | 20 |
| G11F | TAATACTAGAAGACGCCTCAC | 7876 | 21 |
| G11R | AGTGGTCATGGGCTRGGRTC | 8845 | 20 |
| G12F | GAAGTWGCSGTHGCAATAAT | 8738 | 20 |
| G12R | GATTGGAAGTCACTTGTACT | 9616 | 20 |
| G13F | TGATACTGACAYTTYGTTG | 9529 | 19 |
| G13R | GTARTGTTCACAGGGCTA | 10218 | 19 |
| G14F | GAGCAGAATAAGGGAGTTAGT | 10008 | 21 |
| G14R | GGRGCYTCTACRTGTGCTT | 10928 | 19 |
| G15F | ATTACMCTDCTCACCTCMCT | 10667 | 20 |
| G15R | TCCCCACATAATTTCCGGTT | 11730 | 19 |
| G16F | GCRGGYTACTCVCTCTAC | 11590 | 18 |
| G16R | ATGTCBCCNACTCGGTTG | 12508 | 18 |
| G17F | GCATTCTTCATTAGCCTHC | 12118 | 19 |
| G17R | GGTTATGGCTGTTAATTCCAC | 13494 | 21 |
| G18F | AGATGCYATTATTGAAGC | 13165 | 18 |
| G18R | TTGCCATTATTGTTCTTGTAG | 14405 | 21 |
| G19F | AGCAGCAAARTANGGNGT | 14239 | 18 |
| G19R | TTHAGKCCGGCDGGGTT | 15015 | 17 |
| G20F | ACACGATTCTTYGCNTTYCACTT | 14922 | 23 |
| G20R | GTTGGTGGTCTCTTACTASA | 15922 | 20 |
| G21F | GAAGATCGGRGGTTAAATYCC | 15578 | 21 |
| G21R | GGGTTTGACAAGGATARCAGG | 16396 | 21 |
| G22F | CTCCTCRTTACYCYACATGCC | 16153 | 21 |
| G22R | GAGCATAGTGGGGTATCTAATC | 504 | 22 |
