# Supplementary material for: Four new complete mitochondrial genomes of Gobioninae fishes (Teleostei: Cyprinidae) and their phylogenetic implications
Source: PeerJ. 2024 Jan 19;12:e16632. doi: 10.7717/peerj.16632 (PMC10802160; doi:10.7717/peerj.16632)
Supplement: Supplemental Information 7 [file peerj-12-16632-s007.doc]

TABLE S3 Different partition schemes of the PCGs in the phylogenetic analysis.

| Subset | Best Model | # sites | subset id | Partition names |
| --- | --- | --- | --- | --- |
| 1 | GTR+I+G | 1618 | de23b519473e48f3a742375813b6506b | cytb_codon1,nad4L_codon1, atp6_codon1, nad1_codon1,nad4_codon1, nad3_codon1 |
| 2 | GTR+I+G | 1311 | eae1a80d17809da07185b94d98435af1 | nad6_codon2,atp6_codon2, nad3_codon2, nad1_codon2,nad4_codon2 |
| 3 | GTR+G | 682 | 49f264605c5842e5d97b5a59aaeabfd3 | nad2_codon3,nad1_codon3 |
| 4 | GTR+I+G | 1017 | 3f637d95cba269d684063d5cebc3435c | atp8_codon1,nad2_codon1, nad5_codon1 |
| 5 | GTR+I+G | 1017 | 01dcfba7192cd1fea656f30d0d5a2386 | nad2_codon2,nad5_codon2, atp8_codon2 |
| 6 | TIMEF+I+G | 519 | 49ade25470c831ca878109cc37799ed1 | cox1_codon1 |
| 7 | TVM+I+G | 1501 | 065264d74bcc799db4fba76a00cd8f27 | cox3_codon2,cox1_codon2, cox2_codon2, nad4L_codon2,cytb_codon2 |
| 8 | GTR+I+G | 1076 | 0b1de142194098e27dee595169431e89 | cox1_codon3,atp8_codon3, cox2_codon3, cox3_codon3 |
| 9 | TRNEF+I+G | 502 | 3956ab96dc8af384bb64e7c8e5d59353 | cox3_codon1,cox2_codon1 |
| 10 | GTR+I+G | 1516 | feedfc808f2d887d5bb366ffc446f5db | atp6_codon3,nad4L_codon3, nad5_codon3, nad4_codon3,nad3_codon3 |
| 11 | GTR+I+G | 173 | c460792fcd7343938a72f2e162d8c323 | nad6_codon1 |
| 12 | GTR+I+G | 173 | 7bd6c9de3e0771578dc14faf78406e4f | nad6_codon3 |
| 13 | GTR+I+G | 382 | 6991826a3a17af726929451c978dff7d | cytb_codon3 |
