# Supplementary material for: Four new complete mitochondrial genomes of Gobioninae fishes (Teleostei: Cyprinidae) and their phylogenetic implications
Source: PeerJ. 2024 Jan 19;12:e16632. doi: 10.7717/peerj.16632 (PMC10802160; doi:10.7717/peerj.16632)
Supplement: Supplemental Information 9 [file peerj-12-16632-s009.doc]

TABLE S5 The nucleotide composition, AT skew and GC skew of protein coding genes of the mitochondrial genomes in this study.

|  | PCGs | A+T（%） | AT skew | GC skew | Pos #1 | A+T | AT skew | GC skew | Pos #2 | A+T | AT skew | GC skew | Pos #3 | A+T | AT skew | GC skew |
| --- | --- | --- | --- | --- | --- | --- | --- | --- | --- | --- | --- | --- | --- | --- | --- | --- |
| *Abbottina binhi* | 11415 | 55.45 | 0.002 | -0.252 | 3805 | 48.12 | 0.128 | -0.001 | 3805 | 58.82 | -0.376 | -0.331 | 3805 | 59.42 | 0.273 | -0.491 |
| *Abbottina obtusirostris* | 11414 | 55.55 | 0.002 | -0.253 | 3805 | 48.30 | 0.127 | -0.001 | 3805 | 58.87 | -0.377 | -0.333 | 3804 | 59.46 | 0.276 | -0.493 |
| *Abbottina rivularis* | 11415 | 55.42 | 0.002 | -0.251 | 3805 | 48.04 | 0.127 | -0.001 | 3805 | 58.82 | -0.375 | -0.332 | 3805 | 59.40 | 0.274 | -0.489 |
| *Acanthogobio guentheri* | 11415 | 55.21 | -0.038 | -0.206 | 3805 | 47.60 | 0.096 | 0.015 | 3805 | 58.90 | -0.378 | -0.326 | 3805 | 59.13 | 0.194 | -0.367 |
| *Belligobio nummifer* | 11415 | 55.73 | -0.014 | -0.240 | 3805 | 47.88 | 0.108 | 0.006 | 3805 | 58.92 | -0.375 | -0.329 | 3805 | 60.39 | 0.240 | -0.472 |
| *Biwia springeri* | 11412 | 56.69 | -0.008 | -0.243 | 3804 | 48.24 | 0.114 | 0.003 | 3804 | 58.96 | -0.379 | -0.325 | 3804 | 62.85 | 0.247 | -0.493 |
| *Biwia zezera* | 11415 | 57.26 | -0.020 | -0.236 | 3805 | 49.01 | 0.095 | 0.022 | 3805 | 59.13 | -0.377 | -0.329 | 3805 | 63.63 | 0.224 | -0.493 |
| *Coreius guichenoti* | 11415 | 57.72 | 0.020 | -0.276 | 3805 | 48.86 | 0.125 | -0.003 | 3805 | 58.87 | -0.376 | -0.333 | 3805 | 65.44 | 0.297 | -0.612 |
| *Coreius heterodon* | 11415 | 60.89 | -0.008 | -0.258 | 3805 | 50.28 | 0.103 | 0.017 | 3805 | 59.16 | -0.378 | -0.331 | 3805 | 73.25 | 0.216 | -0.658 |
| *Gnathopogon imberbis* | 11412 | 57.24 | -0.022 | -0.221 | 3804 | 48.69 | 0.109 | 0.016 | 3804 | 58.99 | -0.377 | -0.331 | 3804 | 64.04 | 0.204 | -0.436 |
| *Gnathopogon nicholsi* | 11415 | 56.94 | -0.036 | -0.209 | 3805 | 48.99 | 0.090 | 0.023 | 3805 | 58.90 | -0.377 | -0.331 | 3805 | 62.94 | 0.186 | -0.394 |
| *Gnathopogon polytaenia* | 11415 | 56.01 | -0.044 | -0.190 | 3805 | 48.54 | 0.101 | 0.019 | 3805 | 58.90 | -0.378 | -0.325 | 3805 | 60.60 | 0.163 | -0.324 |
| *Gnathopogon strigatus* | 11412 | 56.76 | -0.031 | -0.222 | 3804 | 48.37 | 0.107 | 0.016 | 3804 | 59.12 | -0.378 | -0.330 | 3804 | 62.80 | 0.190 | -0.433 |
| *Gnathopogon taeniellus* | 11415 | 56.16 | -0.040 | -0.199 | 3805 | 48.62 | 0.097 | 0.023 | 3805 | 58.98 | -0.377 | -0.330 | 3805 | 60.89 | 0.177 | -0.352 |
| *Gobio acutipinnatus* | 11415 | 54.20 | -0.046 | -0.200 | 3805 | 47.04 | 0.094 | 0.015 | 3805 | 58.92 | -0.377 | -0.326 | 3805 | 56.64 | 0.182 | -0.343 |
| *Gobio cynocephalus* | 11415 | 55.97 | -0.022 | -0.224 | 3805 | 47.81 | 0.107 | 0.008 | 3805 | 59.13 | -0.376 | -0.330 | 3805 | 60.97 | 0.220 | -0.424 |
| *Gobio macrocephalus* | 11415 | 56.54 | -0.016 | -0.238 | 3805 | 48.07 | 0.107 | 0.009 | 3805 | 59.03 | -0.375 | -0.330 | 3805 | 62.52 | 0.229 | -0.480 |
| *Gobio rivuloides* | 11414 | 54.96 | -0.047 | -0.190 | 3805 | 47.49 | 0.089 | 0.026 | 3805 | 58.90 | -0.381 | -0.324 | 3804 | 58.49 | 0.178 | -0.330 |
| *Gobiobotia brevibarba* | 11418 | 54.48 | -0.034 | -0.205 | 3806 | 48.24 | 0.103 | 0.020 | 3806 | 58.88 | -0.378 | -0.329 | 3806 | 56.33 | 0.208 | -0.354 |
| *Gobiobotia filifer* | 11416 | 54.80 | -0.071 | -0.180 | 3806 | 47.64 | 0.082 | 0.022 | 3806 | 58.83 | -0.374 | -0.329 | 3804 | 57.94 | 0.112 | -0.285 |
| *Gobiobotia macrocephala* | 11415 | 54.07 | -0.003 | -0.223 | 3805 | 47.67 | 0.116 | 0.016 | 3805 | 58.58 | -0.377 | -0.334 | 3805 | 55.95 | 0.288 | -0.401 |
| *Gobiobotia naktongensis* | 11415 | 56.66 | -0.008 | -0.234 | 3805 | 48.23 | 0.116 | 0.012 | 3805 | 58.98 | -0.379 | -0.332 | 3805 | 62.79 | 0.246 | -0.469 |
| *Gobiobotia pappenheimi* | 11415 | 56.40 | -0.012 | -0.234 | 3805 | 47.94 | 0.118 | 0.008 | 3805 | 59.11 | -0.379 | -0.333 | 3805 | 62.16 | 0.237 | -0.458 |
| *Gobiocypris rarus* | 11415 | 57.17 | -0.044 | -0.220 | 3805 | 48.78 | 0.101 | 0.014 | 3805 | 59.05 | -0.378 | -0.327 | 3805 | 63.68 | 0.155 | -0.428 |
| *Hemibarbus barbus* | 11415 | 55.30 | -0.010 | -0.243 | 3805 | 47.60 | 0.115 | 0.001 | 3805 | 58.92 | -0.374 | -0.332 | 3805 | 59.37 | 0.250 | -0.468 |
| *Hemibarbus labeo* | 11415 | 55.60 | -0.011 | -0.242 | 3805 | 47.57 | 0.110 | 0.006 | 3805 | 58.92 | -0.374 | -0.331 | 3805 | 60.32 | 0.248 | -0.478 |
| *Hemibarbus longirostris* | 11424 | 53.48 | -0.065 | -0.195 | 3808 | 47.51 | 0.103 | 0.006 | 3808 | 58.64 | -0.378 | -0.330 | 3808 | 54.31 | 0.126 | -0.302 |
| *Hemibarbus maculatus* | 11412 | 55.29 | -0.013 | -0.241 | 3804 | 47.29 | 0.117 | 0.000 | 3804 | 58.91 | -0.376 | -0.329 | 3804 | 59.67 | 0.243 | -0.467 |
| *Hemibarbus medius* | 11412 | 55.63 | -0.009 | -0.251 | 3804 | 47.77 | 0.118 | -0.001 | 3804 | 58.86 | -0.375 | -0.332 | 3804 | 60.28 | 0.248 | -0.497 |
| *Hemibarbus mylodon* | 11421 | 55.47 | -0.048 | -0.221 | 3807 | 47.99 | 0.097 | 0.009 | 3807 | 58.84 | -0.376 | -0.324 | 3807 | 59.57 | 0.160 | -0.414 |
| *Hemibarbus umbrifer* | 11412 | 55.62 | -0.010 | -0.250 | 3804 | 47.74 | 0.117 | 0.000 | 3804 | 58.91 | -0.374 | -0.332 | 3804 | 60.20 | 0.246 | -0.494 |
| *Microphysogobio chinssuensis* | 11415 | 56.27 | -0.002 | -0.238 | 3805 | 47.94 | 0.123 | -0.001 | 3805 | 59.11 | -0.379 | -0.324 | 3805 | 61.76 | 0.261 | -0.468 |
| *Ladislavia taczanowskii* | 11415 | 54.84 | -0.032 | -0.225 | 3805 | 48.04 | 0.105 | 0.016 | 3805 | 58.63 | -0.381 | -0.332 | 3805 | 57.84 | 0.208 | -0.416 |
| *Microphysogobio alticorpus* | 11412 | 56.05 | 0.001 | -0.238 | 3804 | 48.61 | 0.109 | 0.014 | 3804 | 58.99 | -0.373 | -0.333 | 3804 | 60.54 | 0.278 | -0.468 |
| *Microphysogobio amurensis* | 11418 | 55.95 | -0.002 | -0.240 | 3806 | 47.98 | 0.120 | 0.001 | 3806 | 59.01 | -0.376 | -0.326 | 3806 | 60.85 | 0.265 | -0.470 |
| *Microphysogobio brevirostris* | 11415 | 56.56 | -0.002 | -0.246 | 3805 | 48.36 | 0.117 | 0.003 | 3805 | 58.79 | -0.377 | -0.329 | 3805 | 62.52 | 0.259 | -0.496 |
| *Microphysogobio chenhsienensis* | 11415 | 58.17 | -0.027 | -0.223 | 3805 | 49.49 | 0.090 | 0.032 | 3805 | 59.08 | -0.378 | -0.323 | 3805 | 65.94 | 0.200 | -0.480 |
| *Microphysogobio elongata* | 11414 | 56.68 | -0.008 | -0.239 | 3805 | 48.30 | 0.125 | -0.005 | 3805 | 59.21 | -0.379 | -0.327 | 3804 | 62.51 | 0.240 | -0.467 |
| *Microphysogobio fukiensis* | 11409 | 56.97 | 0.015 | -0.263 | 3803 | 48.04 | 0.117 | 0.002 | 3803 | 59.16 | -0.374 | -0.328 | 3803 | 63.71 | 0.299 | -0.568 |
| *Microphysogobio jeoni* | 11415 | 55.94 | -0.004 | -0.241 | 3805 | 47.91 | 0.118 | 0.001 | 3805 | 59.03 | -0.378 | -0.329 | 3805 | 60.87 | 0.262 | -0.471 |
| *Microphysogobio kiatingensis* | 11430 | 56.82 | 0.012 | -0.254 | 3810 | 48.19 | 0.113 | 0.010 | 3810 | 59.21 | -0.374 | -0.324 | 3810 | 63.07 | 0.296 | -0.547 |
| *Microphysogobio koreensis* | 11415 | 56.07 | -0.003 | -0.242 | 3805 | 48.12 | 0.115 | 0.003 | 3805 | 59.00 | -0.376 | -0.327 | 3805 | 61.08 | 0.263 | -0.479 |
| *Microphysogobio liaohensis* | 11415 | 56.48 | -0.009 | -0.239 | 3805 | 48.36 | 0.111 | 0.010 | 3805 | 59.05 | -0.379 | -0.327 | 3805 | 62.02 | 0.248 | -0.482 |
| *Microphysogobio longidorsalis* | 11412 | 55.49 | -0.021 | -0.223 | 3804 | 48.08 | 0.111 | 0.005 | 3804 | 58.94 | -0.380 | -0.327 | 3804 | 59.44 | 0.230 | -0.410 |
| *Microphysogobio rapidus* | 11415 | 55.62 | -0.006 | -0.235 | 3805 | 47.99 | 0.122 | -0.001 | 3805 | 58.98 | -0.379 | -0.326 | 3805 | 59.89 | 0.260 | -0.446 |
| *Microphysogobio tafangensis* | 11412 | 54.28 | -0.002 | -0.240 | 3804 | 47.69 | 0.111 | 0.009 | 3804 | 58.94 | -0.377 | -0.327 | 3804 | 56.20 | 0.295 | -0.456 |
| *Microphysogobio yaluensis* | 11412 | 55.98 | -0.016 | -0.224 | 3804 | 47.84 | 0.121 | 0.002 | 3804 | 59.10 | -0.379 | -0.325 | 3804 | 61.01 | 0.229 | -0.419 |
| *Paracanthobrama guichenoti* | 11415 | 60.02 | -0.035 | -0.237 | 3805 | 50.57 | 0.086 | 0.025 | 3805 | 59.13 | -0.377 | -0.327 | 3805 | 70.35 | 0.165 | -0.548 |
| *Paraleucogobio notacanthus* | 11415 | 56.50 | -0.028 | -0.223 | 3805 | 48.23 | 0.110 | 0.014 | 3805 | 59.08 | -0.376 | -0.331 | 3805 | 62.21 | 0.196 | -0.430 |
| *Platysmacheilus exiguus* | 11415 | 56.73 | 0.008 | -0.254 | 3805 | 48.17 | 0.114 | 0.007 | 3805 | 59.19 | -0.377 | -0.328 | 3805 | 62.84 | 0.290 | -0.537 |
| *Platysmacheilus longibarbatus* | 11424 | 56.20 | -0.002 | -0.241 | 3808 | 48.42 | 0.107 | 0.012 | 3808 | 58.98 | -0.375 | -0.329 | 3808 | 61.19 | 0.271 | -0.486 |
| *Platysmacheilus nudiventris* | 11415 | 57.23 | -0.020 | -0.240 | 3805 | 48.57 | 0.102 | 0.015 | 3805 | 59.11 | -0.378 | -0.325 | 3805 | 64.02 | 0.217 | -0.509 |
| *Pseudogobio esocinus* | 11418 | 56.83 | -0.017 | -0.232 | 3806 | 48.06 | 0.113 | 0.006 | 3806 | 58.93 | -0.375 | -0.336 | 3806 | 63.50 | 0.216 | -0.453 |
| *Pseudogobio guilinensis* | 11415 | 57.60 | 0.003 | -0.260 | 3805 | 48.54 | 0.116 | 0.001 | 3805 | 58.84 | -0.377 | -0.333 | 3805 | 65.41 | 0.259 | -0.561 |
| *Pseudogobio vaillanti* | 11415 | 56.67 | -0.011 | -0.240 | 3805 | 47.94 | 0.114 | -0.002 | 3805 | 58.79 | -0.379 | -0.334 | 3805 | 63.29 | 0.237 | -0.473 |
| *Pseudopungtungia nigra* | 11415 | 57.71 | -0.034 | -0.223 | 3805 | 49.12 | 0.097 | 0.027 | 3805 | 58.71 | -0.376 | -0.335 | 3805 | 65.31 | 0.174 | -0.455 |
| *Pseudopungtungia tenuicorpus* | 11415 | 57.06 | -0.039 | -0.214 | 3805 | 48.78 | 0.097 | 0.030 | 3805 | 58.76 | -0.372 | -0.333 | 3805 | 63.63 | 0.166 | -0.423 |
| *Pseudorasbora elongata* | 11415 | 60.50 | -0.021 | -0.242 | 3805 | 50.07 | 0.095 | 0.023 | 3805 | 59.11 | -0.373 | -0.334 | 3805 | 72.33 | 0.185 | -0.582 |
| *Pseudorasbora interrupta* | 11415 | 59.67 | -0.042 | -0.212 | 3805 | 50.14 | 0.078 | 0.048 | 3805 | 59.03 | -0.375 | -0.329 | 3805 | 69.83 | 0.153 | -0.481 |
| *Pseudorasbora parva* | 11418 | 58.76 | -0.021 | -0.228 | 3806 | 49.89 | 0.091 | 0.033 | 3806 | 59.04 | -0.376 | -0.329 | 3806 | 67.34 | 0.207 | -0.501 |
| *Pseudorasbora pumila* | 11418 | 59.43 | -0.030 | -0.233 | 3806 | 50.00 | 0.094 | 0.031 | 3806 | 59.17 | -0.374 | -0.332 | 3806 | 69.13 | 0.174 | -0.529 |
| *Pungtungia herzi* | 11418 | 57.90 | -0.032 | -0.220 | 3806 | 49.13 | 0.104 | 0.022 | 3806 | 58.85 | -0.374 | -0.335 | 3806 | 65.71 | 0.172 | -0.442 |
| *Rhinogobio cylindricus* | 11415 | 58.00 | 0.003 | -0.265 | 3805 | 48.80 | 0.106 | 0.004 | 3805 | 58.87 | -0.371 | -0.333 | 3805 | 66.33 | 0.261 | -0.593 |
| *Rhinogobio nasutus* | 11415 | 58.13 | 0.001 | -0.262 | 3805 | 49.09 | 0.107 | 0.005 | 3805 | 58.95 | -0.378 | -0.328 | 3805 | 66.36 | 0.258 | -0.584 |
| *Rhinogobio typus* | 11415 | 58.20 | 0.007 | -0.269 | 3805 | 48.83 | 0.115 | -0.002 | 3805 | 58.98 | -0.373 | -0.331 | 3805 | 66.78 | 0.265 | -0.604 |
| *Rhinogobio ventralis* | 11415 | 58.03 | 0.004 | -0.267 | 3805 | 48.83 | 0.108 | 0.003 | 3805 | 58.84 | -0.371 | -0.333 | 3805 | 66.41 | 0.259 | -0.595 |
| *Romanogobio tenuicorpus* | 11415 | 55.94 | -0.003 | -0.239 | 3805 | 47.99 | 0.120 | 0.002 | 3805 | 59.00 | -0.376 | -0.326 | 3805 | 60.84 | 0.263 | -0.467 |
| *Sarcocheilichthys biwaensis* | 11409 | 54.02 | -0.024 | -0.228 | 3803 | 47.36 | 0.116 | 0.012 | 3803 | 58.74 | -0.379 | -0.333 | 3803 | 55.96 | 0.230 | -0.415 |
| *Sarcocheilichthys davidi* | 11412 | 55.66 | -0.011 | -0.251 | 3804 | 48.21 | 0.124 | 0.003 | 3804 | 58.96 | -0.374 | -0.338 | 3804 | 59.81 | 0.237 | -0.489 |
| *Sarcocheilichthys kiangsiensis* | 11411 | 54.86 | -0.009 | -0.247 | 3803 | 48.09 | 0.126 | 0.003 | 3804 | 58.81 | -0.376 | -0.334 | 3804 | 57.68 | 0.253 | -0.470 |
| *Sarcocheilichthys lacustris* | 11415 | 56.36 | -0.002 | -0.253 | 3805 | 48.30 | 0.128 | -0.007 | 3805 | 58.87 | -0.376 | -0.338 | 3805 | 61.92 | 0.252 | -0.496 |
| *Sarcocheilichthys nigripinnis* | 11412 | 56.01 | -0.015 | -0.245 | 3804 | 48.53 | 0.118 | 0.007 | 3804 | 58.89 | -0.375 | -0.335 | 3804 | 60.62 | 0.228 | -0.481 |
| *Sarcocheilichthys parvus* | 11415 | 56.03 | -0.005 | -0.257 | 3805 | 48.49 | 0.121 | 0.003 | 3805 | 58.87 | -0.374 | -0.333 | 3805 | 60.74 | 0.253 | -0.519 |
| *Sarcocheilichthys sinensis* | 11418 | 56.60 | -0.004 | -0.251 | 3806 | 48.19 | 0.129 | -0.005 | 3806 | 58.75 | -0.377 | -0.336 | 3806 | 62.87 | 0.241 | -0.499 |
| *Sarcocheilichthys variegatus* | 11415 | 56.20 | -0.011 | -0.252 | 3805 | 48.62 | 0.120 | 0.004 | 3805 | 58.87 | -0.375 | -0.335 | 3805 | 61.10 | 0.235 | -0.500 |
| *Saurogobio dabryi* | 11415 | 55.98 | -0.023 | -0.253 | 3805 | 48.02 | 0.099 | 0.014 | 3805 | 58.79 | -0.377 | -0.332 | 3805 | 61.13 | 0.222 | -0.525 |
| *Saurogobio dumerili* | 11415 | 55.02 | -0.003 | -0.270 | 3805 | 47.36 | 0.113 | 0.003 | 3805 | 58.66 | -0.377 | -0.330 | 3805 | 59.03 | 0.277 | -0.561 |
| *Saurogobio gracilicaudatus* | 11415 | 57.11 | -0.035 | -0.232 | 3805 | 49.01 | 0.072 | 0.036 | 3805 | 58.95 | -0.380 | -0.325 | 3805 | 63.36 | 0.204 | -0.502 |
| *Saurogobio gymnocheilus* | 11415 | 55.58 | -0.014 | -0.261 | 3805 | 48.44 | 0.103 | 0.013 | 3805 | 58.79 | -0.377 | -0.332 | 3805 | 59.50 | 0.251 | -0.538 |
| *Saurogobio xiangjiangensis* | 11415 | 56.05 | -0.006 | -0.265 | 3805 | 47.94 | 0.107 | 0.005 | 3805 | 58.84 | -0.377 | -0.330 | 3805 | 61.37 | 0.261 | -0.559 |
| *Squalidus argentatus* | 11415 | 55.80 | 0.017 | -0.275 | 3805 | 47.36 | 0.129 | -0.010 | 3805 | 58.87 | -0.374 | -0.334 | 3805 | 61.16 | 0.308 | -0.572 |
| *Squalidus chankaensis* | 11415 | 54.96 | 0.002 | -0.267 | 3805 | 47.20 | 0.134 | -0.014 | 3805 | 58.76 | -0.376 | -0.336 | 3805 | 58.92 | 0.273 | -0.524 |
| *Squalidus gracilis* | 11412 | 55.07 | 0.000 | -0.265 | 3804 | 47.21 | 0.134 | -0.013 | 3804 | 58.78 | -0.375 | -0.333 | 3804 | 59.23 | 0.267 | -0.523 |
| *Squalidus japonicus* | 11415 | 51.51 | -0.032 | -0.215 | 3805 | 46.07 | 0.124 | 0.001 | 3805 | 58.71 | -0.380 | -0.330 | 3805 | 49.75 | 0.234 | -0.351 |
| *Squalidus mantschuricus* | 11415 | 55.84 | 0.007 | -0.263 | 3805 | 47.44 | 0.127 | -0.008 | 3805 | 58.87 | -0.375 | -0.333 | 3805 | 61.21 | 0.281 | -0.535 |
| *Squalidus wolterstorffi* | 11412 | 55.82 | 0.013 | -0.273 | 3804 | 47.37 | 0.132 | -0.014 | 3804 | 58.73 | -0.375 | -0.334 | 3804 | 61.36 | 0.293 | -0.561 |
| *Xenophysogobio boulengeri* | 11415 | 58.06 | -0.001 | -0.244 | 3805 | 49.43 | 0.103 | 0.018 | 3805 | 58.90 | -0.378 | -0.331 | 3805 | 65.83 | 0.259 | -0.526 |
| *Xenophysogobio nudicorpa* | 11415 | 57.31 | -0.005 | -0.230 | 3805 | 48.94 | 0.100 | 0.026 | 3805 | 58.82 | -0.378 | -0.330 | 3805 | 64.18 | 0.256 | -0.482 |
| *Coreoleuciscus splendidus* | 11418 | 58.66 | -0.016 | -0.244 | 3806 | 49.13 | 0.093 | 0.030 | 3806 | 59.04 | -0.373 | -0.334 | 3806 | 67.81 | 0.217 | -0.562 |
| Avg. | 11415 | 56.36 | -0.014 | -0.238 | 3805 | 48.29 | 0.110 | 0.009 | 3805 | 58.93 | -0.376 | -0.330 | 3805 | 61.87 | 0.233 | -0.473 |
