# Supplementary material for: Four new complete mitochondrial genomes of Gobioninae fishes (Teleostei: Cyprinidae) and their phylogenetic implications
Source: PeerJ. 2024 Jan 19;12:e16632. doi: 10.7717/peerj.16632 (PMC10802160; doi:10.7717/peerj.16632)
Supplement: Supplemental Information 10 [file peerj-12-16632-s010.doc]

TABLE S6 The stop codons of the PCGs of the mitochondrial genomes in this study.

| Species | atp6 | atp8 | cox1 | cox2 | cox3 | cytb | nad1 | nad2 | nad3 | nad4 | nad4L | nad5 | nad6 |
| --- | --- | --- | --- | --- | --- | --- | --- | --- | --- | --- | --- | --- | --- |
| *Abbottina binhi* | TAA | TAA | TAA | T | T | T | TAG | T | T | TA | TAA | TAA | TAG |
| *Abbottina obtusirostris* | TAA | TAA | TAA | T | T | T | TAG | T | TA | TA | TAA | TAA | TAG |
| *Abbottina rivularis* | TAA | TAA | TAA | T | T | T | TAG | T | TA | TA | TAA | TAA | TAA |
| *Acanthogobio guentheri* | TAA | TAA | TAA | T | T | T | TAG | T | TA | TA | TAA | TAA | TAG |
| *Belligobio nummifer* | TAA | TAA | TAA | T | T | T | TAG | T | T | TA | TAA | TAA | TAG |
| *Biwia springeri* | TAA | TAA | TAA | T | T | T | TAA | TA | TA | TA | TAA | TAA | TAA |
| *Biwia zezera* | TA | TAA | TAA | T | T | T | TAG | T | T | TA | TAA | TAA | TAG |
| *Coreius guichenoti* | TAA | TAA | TAA | T | T | T | TAA | TA | TA | T | TAA | TAA | TAA |
| *Coreius heterodon* | TAA | TAA | TAA | T | T | T | TAA | TA | TA | TA | TAA | TAA | TAA |
| *Coreoleuciscus splendidus* | TA | TAA | TAA | T | T | T | TAG | T | T | TA | TAA | TAG | TAA |
| *Gnathopogon imberbis* | TAA | TAA | TAA | T | T | T | TAG | T | TA | TA | TAA | TAA | TAA |
| *Gnathopogon nicholsi* | TAA | TAA | TAA | T | T | T | TAA | TAA | T | TAG | TAA | TAG | TAA |
| *Gnathopogon polytaenia* | TAA | TAA | TAA | T | T | C | TAG | TA | T | TA | TAA | TAG | TAG |
| *Gnathopogon strigatus* | TAA | TAA | TAA | T | T | T | TAA | T | TA | TA | TAA | TAA | TAA |
| *Gnathopogon taeniellus* | TAA | TAA | TAA | T | T | T | TAA | T | T | TA | TAA | TAA | TAG |
| *Gobio acutipinnatus* | TAA | TAA | TAA | T | T | T | TAA | T | T | TA | TAA | TAG | TAA |
| *Gobio botiabrevibarba* | TA | TAA | TAA | T | T | T | TAG | T | T | TA | TAA | TAA | TAG |
| *Gobio botiafilifer* | TAA | TAA | TAA | T | T | T | TAA | T | TA | TA | TAA | TAG | TAA |
| *Gobio cynocephalus* | TAA | TAA | TAA | T | T | T | TAA | T | TA | TA | TAA | TAG | TAA |
| *Gobio macrocephalus* | TAA | TAA | TAA | T | T | T | TAG | T | TA | TA | TAA | TAA | TAA |
| *Gobio rivuloides* | TAA | TAA | TAA | T | T | T | TAG | TA | T | TA | TAA | TAA | TAG |
| *Gobiobotia macrocephala* | TAA | TAA | TAA | T | T | T | TAG | T | TA | TA | TAA | TAG | TAA |
| *Gobiobotia naktongensis* | TAA | TAA | TAA | T | T | T | TAA | T | TA | TA | TAA | TAA | TAA |
| *Gobiobotia pappenheimi* | TAA | TAA | TAA | T | T | T | TAA | T | T | TA | TAA | TAA | TAA |
| *Gobiocypris rarus* | TAA | TAA | TAA | T | T | T | TAA | TA | T | TA | TAA | TAG | TAG |
| *Hemibarbus barbus* | TAA | TAA | TAA | T | TA | T | TAG | T | TAG | TAG | TAA | TAA | TAA |
| *Hemibarbus labeo* | TA | TAA | TAA | T | T | T | TAA | T | T | TA | TAA | TAA | TAG |
| *Hemibarbus longirostris* | TAA | TAA | TAA | T | TA | T | TAA | T | T | T | TAA | TAA | TAG |
| *Hemibarbus maculatus* | TAA | TAA | TAA | T | T | T | TA | T | T | TA | TAA | TAA | TAG |
| *Hemibarbus medius* | TA | TAA | TAA | T | T | T | TAA | T | T | TA | TAA | TAA | TAG |
| *Hemibarbus mylodon* | TAA | TAA | TAA | T | T | T | TAG | TA | TA | TA | TAA | TAA | TAA |
| *Hemibarbus umbrifer* | TAA | TAA | TAA | T | T | T | TAG | TA | TA | TA | TAA | TAG | TAG |
| *Microphysogobio chinssuensis* | TAA | TAA | TAA | T | TA | T | TAA | TAG | TAG | TAG | TAA | TAA | TAA |
| *Ladislavia taczanowskii* | TAA | TAA | TAA | T | T | T | TAG | TA | TA | TA | TAA | TAG | TAG |
| *Microphysogobio alticorpus* | TAA | TAA | TAA | T | T | T | TAG | TAA | TA | TA | TAA | TAG | TAG |
| *Microphysogobio amurensis* | TA | TAA | TAA | T | T | T | TAG | TA | TA | TA | TAA | TAA | TAG |
| *Microphysogobio brevirostris* | TAA | TAA | TAA | T | T | T | TAG | TA | TA | TA | TAA | TAG | TAG |
| *Microphysogobio chenhsienensis* | TAA | TAA | TAA | T | T | T | TAG | TA | TA | TA | TAA | TAA | TAG |
| *Microphysogobio elongata* | TAA | TAA | TAA | T | T | T | TAG | TA | T | TA | TA | TAA | TAA |
| *Microphysogobio fukiensis* | TA | TAA | TAA | T | T | T | TAG | TA | TA | TA | TAA | TAG | TAG |
| *Microphysogobio jeoni* | TAA | TAA | TAA | T | T | T | TAG | TA | TA | TA | TAA | TAA | TAG |
| *Microphysogobio kiatingensis* | TA | TAA | TAA | T | T | T | TAG | T | T | TA | TAA | TAA | TAG |
| *Microphysogobio koreensis* | TAA | TAA | TAA | T | T | T | TAG | TA | TA | T | TAA | TAA | TAG |
| *Microphysogobio liaohensis* | TAA | TAA | TAA | T | T | T | TAG | TA | TA | TA | TAA | TAA | TAG |
| *Microphysogobio longidorsalis* | TAA | TAA | TAA | T | T | T | TAG | T | TA | TA | TAA | TAA | TAA |
| *Microphysogobio rapidus* | TAA | TAA | TAA | TAA | T | T | TAG | TA | TA | TA | TAA | TAA | TAG |
| *Microphysogobio tafangensis* | TAA | TAA | TAA | T | T | T | TAG | TA | TA | TA | TAA | TAA | TAA |
| *Microphysogobio yaluensis* | TAA | TAG | TAA | T | T | T | TAG | TA | T | TA | TAA | TAA | TAA |
| *Paracanthobrama guichenoti* | TAA | TAA | AGG | T | T | T | TAG | TA | TA | TA | TAA | TAA | TAA |
| *Paraleucogobio notacanthus* | TAA | TAA | TAA | T | T | T | TAA | TA | TA | TA | TAA | TAA | TAA |
| *Platysmacheilus exiguus* | TAA | TAA | TAA | T | T | T | TAG | TA | TA | TA | TAA | TAA | TAG |
| *Platysmacheilus longibarbatus* | TAA | TAA | TAA | T | T | T | TAG | TA | TA | TA | TAA | TAA | TAA |
| *Platysmacheilus nudiventris* | TAA | TAG | TAA | T | T | T | TAA | TA | TA | T | TAA | TAA | TAA |
| *Pseudogobio esocinus* | TAA | TAA | TAA | T | T | T | TAG | TA | TA | T | TAA | TAA | TAG |
| *Pseudogobio guilinensis* | TAA | TAA | TAA | T | T | T | TAG | TA | TA | TA | TAA | TAA | TAA |
| *Pseudogobio vaillanti* | TAA | TAA | TAA | T | T | T | TAA | TA | TA | TA | TAA | TAA | TAA |
| *Pseudopungtungia nigra* | TAA | TAA | TAA | T | T | T | TAA | TA | TA | TA | TAA | TAA | TAA |
| *Pseudopungtungia tenuicorpus* | TAA | TAA | TAA | T | T | T | TAA | TAA | TA | TA | TAA | TAA | TAA |
| *Pseudorasbora elongata* | TAA | TAG | TAA | T | T | T | TAA | T | TA | TA | TAA | TAG | TAA |
| *Pseudorasbora interrupta* | TAA | TAA | TAA | T | T | T | TAG | TAA | TA | TA | TAA | TAA | TAA |
| *Pseudorasbora parva* | TAA | TAG | TAA | T | TA | T | TAG | T | T | TA | TAA | TAA | TAA |
| *Pseudorasbora pumila* | TAA | TAA | TAA | T | T | T | TAG | TAG | TA | TA | TAA | TAA | TAA |
| *Pungtungia herzi* | TAA | TAA | TAA | T | T | T | TAG | TA | TA | TA | TAA | TAA | TAA |
| *Rhinogobio cylindricus* | TAA | TAA | TAA | T | T | T | TAA | TA | TA | TA | TAA | TAG | TAA |
| *Rhinogobio nasutus* | TAA | TAA | TAA | T | T | T | TAA | TA | TA | TA | TAA | TAG | TAA |
| *Rhinogobio typus* | TAA | TAA | TAA | T | T | T | TAG | TA | TA | TA | TAA | TAG | TAG |
| *Rhinogobio ventralis* | TA | TAG | TAA | T | T | T | TAA | TA | TA | TA | TAA | TAG | TAA |
| *Romanogobio tenuicorpus* | TAA | TAA | TAA | T | T | T | TAA | TA | TA | TA | TAA | TAG | TAA |
| *Sarcocheilichthys biwaensis* | TAA | TAA | TAA | T | T | T | TAG | TA | TA | TA | TAA | TAA | TAG |
| *Sarcocheilichthys davidi* | TAA | TAG | TAA | T | T | T | TAA | TA | TA | TA | TAA | TAA | TAG |
| *Sarcocheilichthys kiangsiensis* | TAA | TAA | TAA | T | T | T | TAG | TA | T | TA | TAA | TAA | TAA |
| *Sarcocheilichthys lacustris* | TAA | TAA | TAA | T | T | T | TAG | TA | T | TA | TAA | TAA | TAG |
| *Sarcocheilichthys nigripinnis* | TAA | TAA | TAA | T | T | T | TAG | T | T | TA | TAA | TAA | TAG |
| *Sarcocheilichthys parvus* | TAA | TAA | TAA | T | T | T | TAG | T | T | T | TAA | TAA | TAA |
| *Sarcocheilichthys sinensis* | TAA | TAA | TAA | T | T | T | TAA | T | TA | T | TAA | TAA | TAG |
| *Sarcocheilichthys variegatus* | TA | TAA | TAA | T | T | T | TAG | T | T | TA | TAA | TAA | TAA |
| *Saurogobio dabryi* | TAA | TAA | TAA | T | T | T | TAG | TA | TA | TA | TAA | TAA | TAG |
| *Saurogobio dumerili* | TAA | TAA | TAA | T | T | T | TAA | TA | TA | TA | TAA | TAG | TAA |
| *Saurogobio gracilicaudatus* | TAA | TAA | TAA | T | T | T | TAG | TA | TA | TA | TAA | TAA | TAG |
| *Saurogobio gymnocheilus* | TAA | TAA | TAA | T | T | T | TAA | TA | T | TA | TAA | TAA | TAA |
| *Saurogobio xiangjiangensis* | TAA | TAA | TAA | T | T | T | TAA | TA | T | TA | TAA | TAA | TAA |
| *Squalidus argentatus* | TAA | TAA | TAA | T | T | T | TAA | TA | TA | TA | TAA | TAG | TAA |
| *Squalidus chankaensis* | TAA | TAA | TAA | T | T | T | TAA | TA | TA | TA | TAA | TAA | TAG |
| *Squalidus gracilis* | TAA | TAA | TAA | T | T | T | TAG | TA | T | TA | TAA | TAG | TAG |
| *Squalidus japonicus* | TAA | TAA | TAA | T | T | T | TAG | TA | T | TA | TAA | TAA | TAA |
| *Squalidus mantschuricus* | TAA | TAA | TAA | T | T | T | TAA | T | T | TA | TAA | TAA | TAA |
| *Squalidus wolterstorffi* | TA | TAA | TAA | T | T | T | TAA | T | T | TA | TAA | TAA | TAG |
| *Xenophysogobio boulengeri* | TAA | TAA | TAA | T | T | T | TAA | T | T | TA | TAA | TAA | TAA |
| *Xenophysogobio nudicorpa* | TAA | TAA | TAA | T | TA | T | TAA | T | TAA | TA | TAA | TAA | TAA |
