# Supplementary material for: Four new complete mitochondrial genomes of Gobioninae fishes (Teleostei: Cyprinidae) and their phylogenetic implications
Source: PeerJ. 2024 Jan 19;12:e16632. doi: 10.7717/peerj.16632 (PMC10802160; doi:10.7717/peerj.16632)
Supplement: Supplemental Information 11 [file peerj-12-16632-s011.doc]

TABLE S7 The RSCU content of four newly sequenced mitochondiral genomes in this study.

| AA | Gobio rivuloides | | *Microphysogobio elongatus* | | *Rhinogobio nasutus* | | *Microphysogobio chinssuensis* | |
| --- | --- | --- | --- | --- | --- | --- | --- | --- |
|  | Count | % | Count | % | Count | % | Count | % |
| Phe (F) | 232 | 6.11 | 228 | 6 | 230 | 6.06 | 229 | 6.03 |
| Leu2 (L2) | 138 | 3.63 | 127 | 3.34 | 149 | 3.92 | 119 | 3.13 |
| Leu1 (L1) | 480 | 12.64 | 496 | 13.06 | 473 | 12.45 | 500 | 13.16 |
| Ile (I) | 266 | 7.01 | 288 | 7.58 | 295 | 7.77 | 286 | 7.53 |
| Met (M) | 179 | 4.71 | 186 | 4.9 | 181 | 4.77 | 187 | 4.92 |
| Val (V) | 252 | 6.64 | 227 | 5.98 | 217 | 5.71 | 230 | 6.06 |
| Ser2 (S2) | 189 | 4.98 | 187 | 4.92 | 186 | 4.9 | 188 | 4.95 |
| Pro (P) | 216 | 5.69 | 215 | 5.66 | 216 | 5.69 | 216 | 5.69 |
| Thr (T) | 289 | 7.61 | 303 | 7.98 | 302 | 7.95 | 295 | 7.77 |
| Ala (A) | 341 | 8.98 | 325 | 8.56 | 333 | 8.77 | 331 | 8.72 |
| Tyr (Y) | 111 | 2.92 | 110 | 2.9 | 115 | 3.03 | 110 | 2.9 |
| His (H) | 105 | 2.77 | 104 | 2.74 | 102 | 2.69 | 102 | 2.69 |
| Gln (Q) | 93 | 2.45 | 95 | 2.5 | 97 | 2.55 | 95 | 2.5 |
| Asn (N) | 117 | 3.08 | 125 | 3.29 | 121 | 3.19 | 126 | 3.32 |
| Lys (K) | 77 | 2.03 | 78 | 2.05 | 78 | 2.05 | 78 | 2.05 |
| Asp (D) | 81 | 2.13 | 79 | 2.08 | 76 | 2 | 79 | 2.08 |
| Glu (E) | 103 | 2.71 | 102 | 2.69 | 102 | 2.69 | 101 | 2.66 |
| Cys (C) | 25 | 0.66 | 25 | 0.66 | 26 | 0.68 | 25 | 0.66 |
| Trp (W) | 121 | 3.19 | 120 | 3.16 | 121 | 3.19 | 122 | 3.21 |
| Arg (R) | 79 | 2.08 | 78 | 2.05 | 76 | 2 | 78 | 2.05 |
| Ser1 (S1) | 56 | 1.47 | 53 | 1.4 | 57 | 1.5 | 52 | 1.37 |
| Gly (G) | 247 | 6.51 | 246 | 6.48 | 245 | 6.45 | 249 | 6.56 |
| codon end in A or T | 2225 | 58.6 | 2378 | 62.63 | 2525 | 66.48 | 2350 | 61.87 |
| codon end in G or T | 1444 | 38.03 | 1284 | 33.82 | 1203 | 31.67 | 1255 | 33.04 |
| Total | 3797 |  | 3797 |  | 3798 |  | 3798 |  |
